# Assessing the direct and spillover protective effectiveness of *Wolbachia*-mediated introgression to combat dengue

**DOI:** 10.1016/j.ebiom.2024.105456

**Published:** 2024-11-29

**Authors:** Jo Yi Chow, Somya Bansal, Borame S.L. Dickens, Pei Ma, Ary Hoffmann, Yoon Ling Cheong, Nazni Wasi Ahmad, Jue Tao Lim

**Affiliations:** a Lee Kong Chian School of Medicine, Nanyang Technological University, Singapore, 308232, Singapore; b Saw Swee Hock School of Public Health, National University of Singapore, Singapore, 117549, Singapore; c Pest and Environmental Research Group, Bio21 Institute, University of Melbourne, Melbourne, VIC, 3010, Australia; d Biomedical Museum Unit, Special Resource Centre, Institute for Medical Research, Jalan Pahang, Kuala Lumpur, 50588, Malaysia; e Medical Entomology Unit, Infectious Disease Research Centre, Institute for Medical Research, Jalan Pahang, Kuala Lumpur, 50588, Malaysia

**Keywords:** *Wolbachia*, Dengue, Vector control, Synthetic control method (SCM), Spillover, Partial interference

## Abstract

**Background:**

Dengue remains a global health challenge with limited treatment options, highlighting the need for effective vector control strategies. The introduction of *Wolbachia pipientis* into *Aedes aegypti* populations has shown success in reducing dengue transmission across global field trials. However, the spillover effectiveness of the technology on untreated areas is not well-known. This study estimates the spillover protective effectiveness (PE) of *Wolbachia*-mediated introgression on dengue.

**Methods:**

We used the synthetic control method (SCM) under assumption of partial interference to evaluate the direct and spillover PEs of *Wolbachia*-mediated introgression in a long-running operational trial of the intervention in Malaysia. Synthetic controls (SCs), which comprise of a weighted sum of non-spillover controls, were constructed for each directly-treated and spillover site in the pre-intervention period to account for historical imbalances in dengue risk and risk trajectories. SCs were compared to directly/spillover-treated sites to estimate the impact of *Wolbachia-*introgression on dengue incidence across each site, calendar year and intervention time. Robustness checks, including visual inspections, root-mean-square error (RMSE) calculations, in-space and in-time placebo checks, and permutation tests, were used to inspect the model's ability in attributing dengue incidence reductions to the *Wolbachia* interventions.

**Findings:**

The direct and spillover PEs of *Wolbachia* on dengue incidence were expressed as a percentage reduction of dengue incidence, or the absolute case reductions, by comparing SCs to actual intervention/spillover sites. Findings indicate a direct reduction in dengue incidence by 64.35% (95% CI: 63.50–66.71, p < 0.05 using permutation tests) in directly treated areas, corresponding to 1802 (95% CI: 1768–1932) cases averted. Meanwhile, spillover effects contributed to a 37.69% (95% CI: 36.45–38.49, p < 0.05) reduction in adjacent non-intervention areas, accounting for 115 (95% CI: 104–132) absolute cases averted. Tracking PEs by intervention time revealed a dose–response relationship, where PEs increased concomitantly with *Wolbachia* frequency. Model checks confirmed the robustness of these results, and ascertained that these PEs were not an artefact of poor control selection, pre-trends in dengue incidence or poor predictive ability of the fitted SCs.

**Interpretation:**

*Wolbachia-*introgression effectively diminished dengue incidence in directly-treated and surrounding spillover regions. This dual effectiveness highlights the potential of *Wolbachia*-infected mosquitoes as a sustainable, cost-effective strategy against dengue.

**Funding:**

This research is hosted by CNRS@CREATE and supported by the National Research Foundation, 10.13039/100020701Prime Minister's Office, Singapore, under its Campus for Research Excellence and Technological Enterprise (CREATE) program, and is funded by the 10.13039/501100011738Lee Kong Chian School of Medicine—Ministry of Education Start-Up Grant. The original Hoffmann et al. (2024) study was funded by the 10.13039/100010269Wellcome Trust Awards 226166, 108508, 202888 and the 10.13039/501100013885Ministry of Health Malaysia NMRR-16-297-28898.

**Research in context**

**Evidence before this study**

While the effectiveness of releasing *Wolbachia*-infected *Aedes aegypti* mosquitoes for dengue control has been studied, most evaluations have focused on directly-intervened sites. The potential for spillover effects—whereby *Wolbachia*-infected mosquitoes impact dengue transmission in untreated neighbouring areas—remains less understood. To identify any existing evidence on the spillover effects of *Wolbachia*-infected *A. aegypti* releases, we conducted a search of PubMed, encompassing all publications from database inception until May 15, 2024. The search strategy included the type of intervention ((*Wolbachia*) OR (“incompatible insect technique”) OR (“sterile insect technique”) OR (“*Wolbachia*-infected”) OR (“*Wolbachia* introgression”) OR (“Introgression”)) and ((“efficacy”) OR (“effectiveness”)) and (“dengue”) as the outcome of interest for our study. This search yielded 117 relevant studies, which were supplemented by consultations with domain experts. Among these, seven studies demonstrated the effectiveness of the *Wolbachia-*introgression strategy in directly reducing dengue incidence in countries like Australia, Indonesia, and Brazil. Two studies showed the direct effect of incompatible insect techniques on *A. aegypti* populations in Singapore and California, USA. Most importantly, no studies directly examining *Wolbachia* spillover effects on dengue incidence in untreated areas were found, exhibiting a gap in the existing *Wolbachia*-intervention literature.

**Added value of this study**

This study addresses a gap in the current understanding of *Wolbachia*-based interventions by specifically investigating their spillover protective effects on dengue incidence. Using the synthetic control method under partial interference, our results show that *Wolbachia* releases can lead to large reductions in dengue incidence not only in targeted release sites, but also in surrounding untreated areas.

**Implications of all the available evidence**

The findings suggest that *Wolbachi*a-infected *A. aegypti* releases have broader epidemiological benefits than previously documented, extending protective effects to adjacent non-release areas. Future *Wolbachia* release programs could consider these spillover effects when designing and deploying *Wolbachia* to optimise the resource’s impact on dengue control.

## Introduction

Dengue fever has emerged as a rapidly escalating global health crisis, with the World Health Organisation (WHO) reporting an unprecedented 6.5 million cases and over 7300 fatalities in 2023 alone.^1^ The disease is now endemic in more than 100 countries, exposing approximately half of the global population to risk.^1^ Given the limited pharmacological treatments and restricted availability of vaccines for dengue,^2^^,^^3^ vector control remains the cornerstone of efforts to curb dengue transmission.^4^ However, as the population of *Aedes aegypti*, the primary dengue vector, decreases, so does the effectiveness of conventional vector control, attributed to a threshold where the few remaining mosquitoes can still maintain the spread of dengue.^5^ This situation stresses the need for innovative approaches to enhance dengue prevention.

One such strategy is the introduction of *Wolbachia pipientis*, a naturally occurring endosymbiotic bacterium, into *A. aegypti* populations, the primary vector responsible for the transmission of dengue fever. This method has demonstrated substantial effectiveness in reducing dengue transmission in areas where it has been implemented.^6^, ^7^, ^8^, ^9^, ^10^, ^11^, ^12^, ^13^, ^14^ Importantly, field trials in regions such as Yogyakarta,^6^ Australia,^15^ Malaysia,^10^^,^^14^^,^^16^ and Singapore^13^^,^^17^ have not only documented direct effects of *Wolbachia*-interventions, but also revealed potential spillover entomological benefits in adjacent areas. For instance, the randomised controlled trial (RCT) in Yogyakarta reported introgression of *Wolbachia*-infected mosquitoes into control areas,^6^ in addition to a long-term field trial in Singapore using incompatible insect technique coupled with sterile insect technique (IIT-SIT), where surrounding regions had spillover mosquito suppression even though these were sites which did not experience IIT-SIT releases.^17^

Despite the field-based nature of *Wolbachia* interventions, the focus of most studies was on assessing its direct impacts on dengue transmission, with broader spillover epidemiological effects often neglected. Crucially, the flight range of *A. aegypti,*^18^^,^^19^ coupled with human movement, can lead to importation or exportation of dengue virus from intervention and control sites. This dynamic can either attenuate the upper limit of the intervention's protective effectiveness (PEs) in directly-treated locations or confer spillover protective effects in untreated locales. Therefore, we hypothesise that *Wolbachia* interventions are subject to partial interference,^20^^,^^21^ a phenomenon where epidemiological benefits, such as reduced dengue transmission, are observed in geographically proximate study sites that benefit indirectly from nearby interventions, but not in those which are sufficiently distant. While extensive field trials and RCTs in dengue-endemic regions such as Singapore,^13^^,^^17^^,^^22^^,^^23^ Indonesia,^6^ Brazil,^7^^,^^8^ and Malaysia^10^^,^^14^ have confirmed the direct epidemiological effectiveness of the *Wolbachia-*introgression approach, no studies have evaluated these interventions under the conditions of partial interference and estimated *Wolbachia*'s spillover protective effectiveness.

Therefore, our study aimed to examine the PEs which *Wolbachia* can confer in untreated areas. Here, we employed a synthetic control (SC) approach which can account for partial interference, enabling us to quantify the spillover effects of *Wolbachia*-introgression in a case study of a long-running operational trial in Selangor, Malaysia.^14^ Our work provides a three-fold contribution, where we (1) developed a quasi-experimental analytical framework tailored to appropriately account for spillover effects from intervention to control sites; (2) re-estimated PEs for directly-treated locations; and (3) present estimates of spillover PEs of the *Wolbachia*-introgression approach in terms of reducing dengue incidence rates. This work provides a basis to ascertain spillover effects of field-based public health interventions and deepens our epidemiological understanding of vector control interventions to inform future strategies for combating dengue and similar vector-borne diseases.

## Methods

### Epidemiological data (Selangor, Malaysia)

Dengue incidence in Selangor was analysed using a dataset from Hoffmann et al. (2024), which documents dengue incidences in 20 intervention sites and 76 control sites between 2014 and 2022. Incidence rates were calculated as the number of dengue cases per 100,000 people in the population at risk, reported on a weekly basis. According to the ‘Case Definitions for Infectious Diseases in Malaysia 2017′,^24^ a dengue diagnosis mandates concurrent clinical and laboratory criteria: clinically, a sudden high fever lasting over two days and accompanied by at least two symptoms, such as headaches, orbital pain, muscle soreness, joint pain, rash, or slight haemorrhagic signs. Laboratory verification involves detecting the NS1 protein, antibodies specific to dengue, the viral genome through PCR, or isolating the virus from bodily samples. Individuals testing positive by these measures are logged into eDengue, the national registry supervised by the District Health Office. The Supplementary information details more information on the operationalised trial.

### Covariate data

Climate and vegetation data were obtained from ERA5 hourly estimates at a resolution of 0.25°·0.25° (approximately 27.5 km), published by the European Centre for Medium-Range Weather Forecasts (ECMWF).^25^ Downscaled rasters of 100 m·100 m were created using Universal Kriging interpolation. Buffers with a radius of 500 m were established for each study site where epidemiological week maximum, average, and minimum values were calculated. The variables used were 2 m temperature (°C), total precipitation sums (mm), relative humidity (%), fraction of cloud cover (0–1), low vegetation cover (0–1), high vegetation cover (0–1), leaf area index for low vegetation (m^2^/m^2^), and leaf area index for high vegetation (m^2^/m^2^).

### Statistics

#### Synthetic control method (SCM) under partial interference

We employed the canonical synthetic control method (SCM), which constructs synthetic controls (SCs) individually for each intervention and spillover site. These SCs were derived as weighted averages of donor (control) sites that did not receive the intervention and were not spillover sites.^26^ To account for partial interference,^21^ we partitioned the original control pool into two groups: ‘spillover sites’ and ‘pure control sites’. Spillover sites are defined here as those within 1500 m of directly-intervened sites. This is a conservative estimate based on meta-analyses on *Aedes* flight ranges, where one analysis showed *Aedes* mosquitoes having an average maximum flight distance of nearly 3000 m^19^ and another indicated that *A. aegypti* flight distances can range from 12 to approximately 2400 m.^18^ A kinship study of *Wolbachia-*infected mosquitoes conducted in Australia after the Cairns *Wolbachia* releases further detected a pair of putative full-sibling *Wolbachia*-infected mosquitoes in ovitraps located 1312 m apart, hypothesised to be mediated by human transport.^15^ These findings support the designation of 1500 m as a conservative estimate, ensuring that spillover sites fall within the mosquito dispersal range to capture the majority of spillover effects while minimising the risk of overestimating the intervention's spread. Sites beyond this range are classified as pure control sites, based on the assumption that their distance from intervention sites makes spillover effects highly unlikely due to the limited mobility of mosquitoes and minimal human movement between distant residential locations.

We denote d=1:J as directly-treated sites, and partition control sites to be $d^{*}=I_{0}\ldots I_{a},k=I_{a\;+\;1},\ldots ,$*I*_A_ where *d∗* are spillover sites and k are pure control sites. Sites are observed for $t=1,\ldots ,t_{j},\ldots ,T$ where $t_{j}$ is the adoption time of direct-intervention/spillover for some site j. Tracking the differences in adoption date for each intervention/spillover site enables us to account for the staggered intervention/spillover adoption dates of for each site. We generated SCs and accounted for partial interference by minimising the difference in dengue incidence rates between the directly-intervened/spillover sites $y_{j,{1\;:\;t}_{j}\;-\;1}$ and the pure control sites $y_{\;-\;j,{1\;:\;t}_{j}\;-\;1}$ in the pre-intervention period ${1:t}_{j}-1$. We imposed that weights sum to one and are non-negative, following the approach to estimate weights in the canonical SCM:^26^

$${\mathrm{argmin}}_{W_{j}}|y_{j}-\sum_{i\in k}\;w_{j,i}y_{i}|,\left\{\sum_{i\in k}\;w_{j,i}=1,w_{j,i}\geq 0\right\}$$

We adapted the methodology from Grossi et al. (2024),^21^ whereby each directly-intervened/spillover site's SC was constructed using only the pure control sites as the donor pool. Weights were estimated in the same manner, i.e. by searching for weights which can minimise the pre-spillover dengue incidence rates of the respective spillover site and the weighted average of pure control pool sites. The use of SCM eliminates the necessity of selecting traditional control sites by balancing the pre-intervention/spillover epidemiological trajectory of the intervention/spillover site and that of the weighted composite of the pure control arm to generate a respective SC for each intervention/spillover site. This enables the generation of a data-driven counterfactual to the release site, and consequentially causal identification of the intervention/spillover effect post-weight estimation.

Protective effectiveness (PE) for the intervention/spillover site j was estimated as the percentage difference in observed dengue incidence rate between the respective intervention/spillover site $\left(y_{j,t}\right)$ and their respective SCs $\left(\sum_{i\in k}\;{\hat{w}}_{j,i}y_{i,t}\right)$ in the post-intervention or post-spillover period:

$$PE_{SCM,j}=\sum_{t\in \left\{t_{j}:T\right\}}\;\frac{\left(\sum_{i\in k}\;{\hat{w}}_{j,i}y_{i,t}\;-\;y_{j,t}\right)}{\sum_{i\in k}\;{\hat{w}}_{j,i}y_{i,t}}\;\times \;100$$

We also estimated the absolute case aversion by defining it as the differences in dengue incidence between an intervention/spillover site and their respective SCs in the post-intervention/spillover period, scaled by the at-risk population size normalised per 100,000 individuals in the intervention/spillover location. This metric estimates the raw number of dengue cases averted by the intervention.

#### Aggregates of protective effectiveness and absolute case aversion

The outcomes, being both temporally and spatially explicit, allowed us to re-aggregate PEs in ways that address potential temporal and spatial heterogeneity in PEs and consider the time needed for *Wolbachia* interventions to take effect. In the preceding sections, $PE_{SCM,j}$ can be considered as aggregations of PEs for a specific site j. We also computed:

(1) **The overall effect of the intervention across all study sites**, as the difference between the sum of actual dengue incidence rates and sum of counterfactual dengue incidence rates across all sites and then divided by the incidence estimated by SCs across all sites. This is calculated from each site-specific start time of intervention to the end of the study's observation period.
(2) **The PE by event-study time**, where we examined changes in PE by intervention sites which had experienced 1–6, 7–12, 13–18, 19–24, and 24+ months of intervention. For each time period, PEs are computed by re-aggregating them based on the time each site has experienced intervention. The observed difference in incidence rates between the directly-intervened or spillover sites and their corresponding SCs were calculated for each time period and expressed as a proportion of the incidence rates estimated by the SC in that time period.
(3) **The PE by calendar-time** where we computed observed difference in incidence and incidence estimated by SCs by calendar year and then divided by the incidence estimated by SCs for the same time frame (i.e. 2018, 2019, …, 2022) for all sites that received intervention in the specific post-intervention year. The PE is calculated as in the preceding section.

We characterised uncertainty in estimated SC weights and consequentially, our estimates of PE/absolute case aversion, by first generating bootstrap samples of dengue incidence rates using the ‘meboot’ package in R, which can create bootstrap timeseries ensembles without assuming stationarity using the Maximum Entropy Bootstrap method. A total of 10,000 synthetic versions of the original timeseries for each intervention, spillover, and pure control site were then created. By applying SCM to each bootstrap timeseries, an empirical distribution of PEs and absolute cases averted could be constructed, from which 95% confidence intervals (95% CI) could be obtained. PEs and absolute cases averted whose 95% CI does not cross 0 were considered significant.

#### Accounting for covariates

We considered two model specifications to calculate SCM weights for intervention/spillover sites to appropriately account for covariates in the SC framework. We took **(1)** all pre-release observations of the outcome variable (dengue incidence rates) without covariates $\left(M_{nc}\right)$; **(2)** the pre-intervention/spillover average of covariates and dengue incidence rate $\left(M_{avg}\right)$.

$M_{avg}$ is motivated by past work arguing that averaging both covariate and dependent variable values lead to constant weightage between both the outcome variable and covariates for weight estimation, providing a balanced representation of historical trends pre-intervention.^27^ $M_{nc}$ serves as a baseline model, representing the standard SCM without covariates and helps us ascertain the impact of covariate adjustment on model performance. Comparisons of RMSE between the models showed that Model 1 outperformed Model 2 in terms of predictive accuracy (Supplementary Tables S3–S5). Consequently, the use of covariates was precluded from additional assessments.

#### Robustness checks

We extensively explored the internal validity of the SCM which included the two different ways to account for covariates for both intervention and spillover locales. We:

(1) Conducted **visual inspection checks** to ascertain the validity of our model by plotting the fitted SCs against the actual intervention and spillover sites in the pre-intervention period to examine the model fit of dengue incidence rates.
(2) Computed the **pre-treatment root-mean-square error (RMSE)** between the observed and SC dengue incidence rates in the pre-intervention period for each directly-intervened/spillover site. The RMSE serves as a measure of fit between the site's actual pre-intervention outcomes and SC predicted outcomes, with lower RMSEs indicating better fits. Sites with RMSEs >100 were excluded from PE aggregation calculations.
(3) Conducted **in-time placebo checks**, where placebo intervention events 104 weeks (2 years) before the actual intervention start date of each directly-treated/spillover site were constructed to ensure that each SC fits well to the pre-intervention data for intervention/spillover sites, and that the measured effects were attributable to *Wolbachia,* rather than to poorly predictive counterfactuals or pre-intervention trends in dengue incidence rates. Using the optimal model specification, we measured the placebo-intervention/spillover effect during the placebo intervention in the pre-intervention period and compared these to the actual post-intervention direct/spillover effects. Each site's PE was considered to have passed the check for that site if its respective time-placebo PE was smaller than the actual PE.
(4) Conducted **in-space placebo checks**, which comprised rerunning the SCM on placebo intervention sites which were sites which (a) never experienced direct *Wolbachia* interventions or (b) not deemed spillover towns in the actual intervention period. We used a donor pool which excluded intervention and spillover sites and constructed SCs for the designated placebo-intervention and placebo-spillover sites using all other never-treated/never-spillover townships and estimated the direct/spillover effects in the post-intervention period. Small placebo-PEs would help ensure that estimated intervention effects were not due to chance in the post-intervention period for actual intervention/spillover sites.
(5) Performed **permutation tests** by randomly reassigning intervention and control towns 1000 times, where spillover towns were defined based on proximity to the randomly assigned pure control town. We re-estimated SCs and the direct and spillover PEs to form the permutation distribution of direct and spillover PEs. Direct/spillover PEs were deemed significant at the 5% level if the estimated direct/spillover PEs were larger than 95% of all permutation PEs estimated using the permutation procedure. This was an alternative method to conduct inference and ascertain statistical significance.

### Ethics

The original study by Hoffmann et al. (2024), from which our data was derived, received ethical clearance from the Malaysian Ministry of Health's Medical Research and Ethics Committee (MREC) and releases were approved following a risk assessment as documented in Nazni et al. (2019).^10^^,^^14^ As our work involves the reanalysis of previously collected data, no further ethical approval was necessary.

### Role of funders

The funders of the study had no role in study design, data collection, data analysis, interpretation, or writing of the report.

## Results

### Summary statistics and intervention

Table 1 summarises the epidemiological and environmental characteristics across the study period (EW 1 2014—EW 26 2022), broken down by intervention, spillover, and pure control sites. *Wolbachia-*introgression was adopted in 20 intervention sites, and taking spillover sites as those which were 1500 m away from intervention sites, there were 11 spillover sites and 65 pure control sites. However, after excluding sites that failed validation criteria (i.e. visual inspection, RMSE checks and placebo tests), there were 14 intervention sites and 5 spillover sites remaining. During the pre-intervention period (175–354 weeks for intervention sites, 288–315 weeks for spillover sites, and 175 weeks for pure control sites), the average dengue incidence rates were 27.0, 27.2, and 34.1 per 100,000 persons in intervention, spillover, and pure control sites respectively. Post-intervention periods, which spanned 90–269 weeks for intervention sites, 129–156 weeks for spillover sites and 269 weeks for pure control sites, observed dengue incidence rates decreased to 5.4, 10.1 and 23.8 per 100,000 persons in intervention, spillover and pure control sites, respectively. The average *Wolbachia* frequency in the intervention sites was 81.9%, which suggested a high rate of successful introgression. Summaries of the environmental characteristics indicate minimal variation across each site type (Table 1).

**Table 1:** Summary of pre- and post-*Wolbachia* release characteristics and environmental covariates in intervention, spillover and pure control sites after exclusion of sites that failed validation criteria (i.e. visual inspection, RMSE checks and placebo tests).

	Intervention		Spillover		Pure control
	Actual	SC	Actual	SC	
Study characteristics					
Study dates	EW 1 2014–EW 26 2022				
Baseline control measures	Larval source reduction, dengue awareness programs, thermal fogging^e^				
*Wolbachia-*intervention type	Introgression		–		–
Number of sites	14		5		65
At-risk population	116,918		17,775		242,271
Pre-intervention period (weeks)^a^	175–354		288–315		175
Post-intervention period (weeks)^a^	90–269		129–156		269
Intervention time (weeks)^b^	163.87		150.60		269
*Wolbachia* frequency^c^	81.9%		–		–
Dengue incidence rates (pre-intervention)^d^	27.0 (20.6)	25.9 (14.7)	27.2 (13.1)	27.8 (9.8)	34.1 (31.7)
Dengue incidence rates (post-intervention)^d^	5.4 (3.6)	15.2 (10.6)	10.1 (8.4)	16.2 (7.4)	23.8 (25.4)
Covariates^d^					
Temperature (°C)	26.79 (0.77)	26.89 (0.76)	26.79 (0.77)	26.88 (0.77)	26.80 (0.77)
2 m temperature (°C)	27.48 (0.82)	27.45 (0.74)	27.57 (0.77)	27.57 (0.75)	27.33 (0.87)
Total precipitation sums (mm)	0.29 (0.21)	0.28 (0.20)	0.28 (0.20)	0.28 (0.20)	0.30 (0.21)
Relative humidity (%)	79.57 (4.61)	79.46 (4.55)	79.21 (4.40)	78.96 (4.51)	80.17 (4.83)
Fraction of cloud cover (0–1)	0.02 (0.03)	0.02 (0.02)	0.01 (0.02)	0.02 (0.02)	0.02 (0.04)
Low vegetation cover (0–1)	0.74 (0.17)	0.76 (0.08)	0.68 (0.18)	0.77 (0.02)	0.74 (0.23)
High vegetation cover (0–1)	0.10 (0.03)	0.12 (0.01)	0.10 (0.03)	0.11 (0.01)	0.11 (0.03)
Leaf area index for low vegetation (m^2^/m^2^)	2.08 (0.56)	2.31 (0.23)	1.92 (0.35)	2.19 (0.16)	2.24 (0.66)
Leaf area index for high vegetation (m^2^/m^2^)	3.46 (0.70)	3.61 (0.34)	3.15 (0.58)	3.52 (0.07)	3.56 (1.01)

The values in parenthesis represent the standard deviations of the average of the characteristics (i.e. dengue incidence or environmental covariates) based on weekly data over the entire observation period.

a Given as the range of pre- and post-intervention lengths in intervention and spillover sites. Pre-intervention periods for intervention sites run from the start of the study to the week before the initial *Wolbachia* release, and for spillover sites, up to the week before the first release at the nearest intervention site. Post-intervention periods begin at the release week and extend to the study's end, both for intervention and corresponding spillover sites. Pre- and post-intervention periods for pure control sites are based on releases for the earliest directly-intervened site (i.e. W22). Please refer to Supplementary Tables S6–S7 for a detailed breakdown of pre- and post-intervention dengue incidences.

b Average across all sites in that category (intervention or spillover).

c Average percentage of *Wolbachia*-infected *A. aegypti* adults, as detected by quantitative PCR (qPCR), relative to the total number of adult mosquitoes captured in ovitraps across all intervention sites, starting from four weeks after the initial *Wolbachia* release in each intervention site. Monitoring becomes irregular once the *Wolbachia*-infection rate in the field population stabilised at or above 80% for more than three consecutive monitoring periods over three months. No *Wolbachia* frequency was measured in spillover sites.

d Average across all sites within that category (intervention, spillover, or pure control).

e Thermal fogging is not conducted in the intervention sites after the start of *Wolbachia*-releases in that site, and is only continued in the pure control and spillover sites.

### Direct and spillover protective effectiveness for *Wolbachia* interventions aggregated by site

We estimated that *Wolbachia* interventions have led to a 64.35% (95% CI: 63.50–66.71, p < 0.05 using permutation tests) decrease in dengue incidence rates for intervention sites and a 37.69% (95% CI: 36.45–38.49, p < 0.05) decrease in dengue incidence rates for spillover sites. This translated to over 1802 (95% CI: 1768–1930) and 115 (95% CI: 104–132) absolute cases averted for intervention and spillover sites respectively (Table 2, Supplementary Tables S8–S9).

**Table 2:** Protective effectiveness (PE) estimates (%) of *Wolbachia* releases on total dengue incidence rates and absolute cases averted aggregated across all intervention (n = 14) and spillover sites (n = 5) that met the validation criteria through placebo tests and RMSE assessment.

	Direct^a^^,^^c^	Spillover^a^^,^^c^
*Wolbachia*-introgression frequency (%)	81.9	b
Aggregated site PE (%)	64.35 (63.50–66.71), p < 0.05	37.69 (36.45–38.49), p < 0.05
Absolute cases averted	1802 (1768–1930)	115 (104–132)

Estimates are calculated using SCM under assumption of partial interference. Numbers in parenthesis represent lower and upper bounds for 95% confidence intervals, estimated using the bootstrapping procedure. Only sites that passed validation criteria (i.e. visual inspection, RMSE checks and placebo tests) were included in the calculation of these aggregates. The p-values reported for the direct and spillover aggregated PEs were computed using permutation tests.

a Indicates that the PE estimate passed in-time placebo checks while.

b No *Wolbachia-*introgression frequency was measured in spillover sites.

c Indicates that it passed the in-space placebo check.

**Fig. 1:**
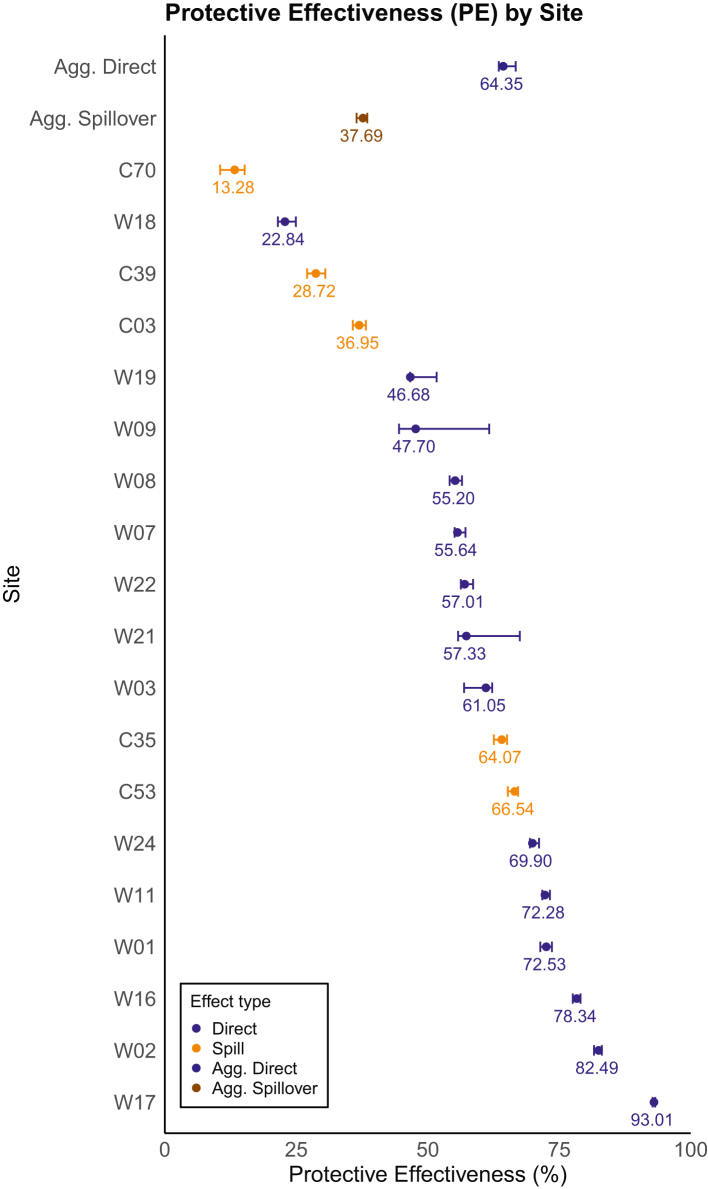

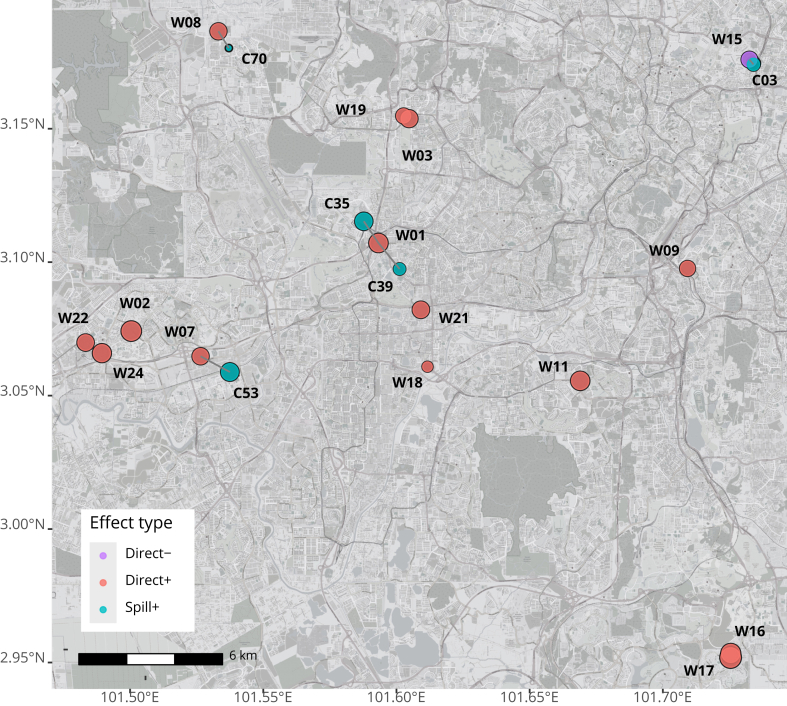
Site-specific protective effectiveness (PE) point estimates and corresponding 95% confidence intervals of *Wolbachia* releases on total dengue incidence rates across intervention (n = 14), spillover sites (n = 5) and on aggregate (Figure 1a), with corresponding spatial location and PEs plotted (Figure 1b). The scale bar in Figure 1b represents a distance of 6 km on the map. Note that for site C03, while its associated intervention site (W15, in purple) did not pass the in-time placebo test, the spillover effects for C03 were robust, passing all validation tests, and thus it is included as a spillover site in the analysis. Sites that did not pass the validation criteria were excluded from these analyses.

Across all sites which were directly-intervened by *Wolbachia*, we estimated PEs for 14 of 20 directly-intervened sites after exclusion of those that did not meet validation criteria (i.e. poor visual fit, model pre-intervention RMSE >100, failed placebo tests; Supplementary Tables S10–S13, Supplementary Figure S1) with PEs ranging from 22.84%–93.01% (Fig. 1a). Spillover PEs were generally smaller compared to direct PEs, but were all positive and ranged from 13.28%–66.54%. Distances to actual intervention sites did not seem to influence estimates of spillover PEs, with large values for spillover PEs estimated even for locations (Fig. 1b: Site C53) which were relatively far (1388 m, Supplementary Table S9) from the closest intervention site (Fig. 1b: Site W07).

### Protective effectiveness aggregated by event time and calendar time

We further examined how time spent as an intervention/spillover site may influence the PE of the intervention and the number of absolute cases averted. Direct PE followed an increasing trend which ranged from 59.45% (95% CI: 58.71–61.23) when sites experienced 1–6 months of *Wolbachia* releases and up to 74.41% (95% CI: 73.80–77.40) when sites experienced more than 2 years of sustained *Wolbachia-*introgression. PEs were higher with greater levels of *Wolbachia* frequency in these sites as well. Similarly, this trend was found for spillover locations albeit with a dip in spillover PEs for sites which were spillover locations for more than 24 months (Table 3).

**Table 3:** Estimates of protective effectiveness (PE) and absolute cases averted for *Wolbachia* releases on total dengue incidence rates across all intervention sites aggregated by event time (top) and calendar time (bottom), with corresponding *Wolbachia* frequencies.

Time period	*Wolbachia*-introgression frequency in direct sites (%)	Direct		Spillover	
		PE (%)	Absolute cases averted	PE (%)	Absolute cases averted
Event time					
1–6 months	71.15	**59.45 (58.71**–**61.23)**	**360 (339–383)**	**36.21 (34.04–37.96)**	**52 (46–58)**
7–12 months	78.87	**54.03 (52.95**–**56.46)**	**181 (169–195)**	**34.83 (32.81**–**36.12)**	**39 (35–45)**
13–18 months	86.68	**64.60 (63.89**–**66.97)**	**240 (227–267)**	**63.24 (62.35–64.01)**	**8 (7–11)**
19–24 months	87.38	**61.00 (60.11–62.88)**	**251 (245–267)**	**72.26 (71.62–72.79)**	**−1 (−1 to −1)**
24 months+	88.67	**74.41 (73.80**–**77.40)**	**770 (766–829)**	**24.62 (23.12–25.50)**	**17 (14–20)**
Calendar time					
2017	70.26	**61.17 (60.51–63.65)**	**108 (103–116)**	–	–
2018	86.22	**29.28 (28.37**–**34.13)**	**157 (148–185)**	–	–
2019	74.15	**67.22 (66.59–68.83)**	**729 (703–769)**	**38.60 (36.39–40.37)**	**43 (39–49)**
2020	80.31	**70.24 (69.34–73.01)**	**451 (436–479)**	**39.50 (38.04–40.48)**	**55 (49–64)**
2021	86.28	**75.95 (75.32–78.53)**	**191 (192–213)**	**53.30 (51.28–54.94)**	**−3 (−5 to −1)**
2022	83.78	**66.17 (65.70**–**68.22)**	**166 (160–181)**	**18.73 (15.95**–**20.71)**	**20 (18–22)**

*Wolbachia-*introgression frequency is determined by the average percentage of the *Wolbachia*-infected *A. aegypti* adults relative to the total number of adult mosquitoes captured in ovitraps across all sites during monitoring phase relative to the start of intervention (introgression), detected by qPCR. Numbers in parenthesis represent 95% confidence intervals estimated by bootstrapping. **Bolded** figures represent significant PEs where 95% confidence intervals do not cross 0. Only sites that passed validation criteria were included in the calculation of these aggregates (i.e. 14 intervention sites and 5 spillover sites).

Estimates of direct PEs by calendar time showed roughly consistent PEs in the range of 29.28 (95% CI: 28.37–34.13) to 75.95 (95% CI: 75.32–78.53), with *Wolbachia*-introgression frequencies maintained at a high 70.26%–86.26% on aggregate. Consequentially, the number of cases averted was consistently large with a range of 108–729 across 2017 to 2022. Similarly, spillover PEs showed an increasing trend with increasing *Wolbachia*-introgression frequency with a slight dip in 2022 (Table 3), potentially due to the study ending at the 26th epidemic week of 2022, making the data less representative of the full year.

### Robustness checks

2 directly-intervened sites and 2 spillover sites did not pass visual inspection and RMSE checks (Supplementary Table S8–S9; Supplementary Figure S1) and were excluded from aggregate PE estimates. Additionally, 4 directly-intervened sites and 5 spillover sites were excluded due to placebo PE estimates exceeding their actual estimates (Supplementary Tables S10–S11). Conversely, all other intervention and spillover sites exhibited a good fit with no significant placebo effects observed during the in-time placebo checks. Meanwhile, in-space placebo tests revealed that 1 pure control site produced a spurious estimate, while all other pure control sites had smaller PEs compared to those from true intervention sites. Notably, the aggregated direct and spillover placebo effects on in-time placebos produced negative estimates (Supplementary Tables S10–S11), whereas the aggregated placebo effects on in-space placebos were reduced by 90% and 30%, respectively (see Supplementary Tables S12–S13). Conducting statistical interference using the permutation tests also demonstrated the statistical significance of our aggregated direct and spillover PE estimates (Supplementary Figure S2), corroborating with the bootstrapping inferential approach.

## Discussion

Using a long-standing operational *Wolbachia*-introgression field trial in Malaysia as a case study, our analysis substantiates the recognised direct PEs of *Wolbachia-*introgression on dengue incidence, estimating a reduction of 64.35% (95% CI: 63.50–66.71, p < 0.05). Crucially, we estimated that *Wolbachia* interventions conferred an indirect benefit to adjacent areas, demonstrating a 37.69% (95% CI: 36.45–38.49, p < 0.05) decrease in dengue incidence rates in spillover sites, preventing approximately 1802 (95% CI: 1768–1930) and 115 (95% CI: 104–132) raw cases at direct and spillover sites, respectively. Robustness checks indicated that estimated PEs were not due to pre-existing epidemiological trends or poorly predictive SC fits.

The direct PE observed aligned closely with the previously published study on Selangor,^14^ which estimated a 62.4% (95% CI: 50.0–71.0%) reduction using a Bayesian model. Comparable results have been reported in global *Wolbachia* trials in locations such as Australia,^11^ Brazil,^9^ Indonesia,^6^ and Singapore.^13^^,^^22^ However, these studies may have encountered challenges that led to underestimated PEs, such as inadvertent *Wolbachia* spread to control sites in the Yogyakarta RCT^6^ and operational disruptions during the COVID-19 pandemic in Brazil,^7^^,^^8^ which diminished *Wolbachia* frequency and consequently, its effectiveness. Similarly, in a preceding study in Selangor,^10^ the proximity of control sites to intervention sites may have resulted in partial interference, complicating the isolation of direct effects. When analysing direct PE without assuming partial interference in the standard SC framework, we observed a slight reduction in PE to 64.19% (95% CI: 59.41–66.40), leading to 1834 (95% CI: 1788–1959) absolute cases averted. This shows the underlying influence of unaccounted spillover effects in diluting PE, underestimating the true impact by 83 cases (Supplementary Tables S8–S9). Furthermore, our estimation of cases averted may be conservative, as it only accounts for designated spillover areas from the original control sites, thereby overlooking the broader impact of *Wolbachia* on surrounding residential zones not initially included in the study. This approach, coupled with our modestly selected spillover distance, suggests that the actual spillover PE of *Wolbachia* may be greater than our current findings indicate.

The biological mechanism underlying the estimated spillover effects can be attributed to the dispersal behaviour of *Wolbachia*-infected mosquitoes and is consistent with past field observations. *Wolbachia-*introgression in non-release areas has been previously demonstrated in a prior RCT of *Wolbachia,*^6^ but its unintended epidemiological benefits was not measured. In Singapore, similar observations were made with the Incompatible Insect Technique-Sterile Insect Technique (IIT-SIT) approach, where short-range suppression of mosquito populations led to mosquito suppression in not-yet-treated sites.^17^ This indicated *Wolbachia-*infected mosquitoes' ability to traverse short distances, invade neighbouring areas and potentially reduce dengue transmission. Reduced dengue infections in intervention sites may also mean that individuals residing in release zones are less likely to act as initial carriers of dengue to control sites. As a result, the risk of seeding new transmission pathways due to infectees’ movement from intervention to control locations can be decreased. This confers a degree of short-range protection to the local population in control sites, minimising the spread of dengue through human movement.

The advantages of *Wolbachia*-based interventions are thereby multi-faceted, as the technology not only reduces dengue incidence in directly-treated areas, but also extends protective benefits to nearby untreated regions. It can also be self-sustaining, as *Wolbachia*-infected mosquitoes autonomously locate and introgress into local wild-type mosquito populations, minimising the need for manual intervention. Moreover, the approach is cost-effective^28^ and potentially cost-saving,^29^ though it necessitates ongoing monitoring to sustain high introgression rates and maintain high protective effects, as illustrated in the Brazil trials.^7^^,^^8^ Fitness plays an important role in the introgression of *Wolbachia*-infected *A. aegypti* populations,^30^^,^^31^ particularly in spillover locations where the frequency of *Wolbachia*-mosquitoes is lower. Prior studies have demonstrated that despite potential fitness costs, *Wolbachia*-infected mosquitoes have shown successful introgression within wild-type populations, driven by cytoplasmic incompatibility.^6^, ^7^, ^8^^,^^10^^,^^14^ This advantage could extend to *Wolbachia* into spillover areas, supporting the establishment and maintenance of the intervention's protective effects in areas without direct releases.

Our study has several strengths. (**1**) We employed a robust SCM model that eliminates biases associated with control selection, even in spillover locations, accompanied by a comprehensive suite of robustness checks that reinforced the internal validity of our findings. (**2**) We accounted for a wide array of environmental covariates, which is often not considered in most prior evaluation studies of *Wolbachia* field trials.^7^^,^^9^, ^10^, ^11^, ^12^^,^^14^ (**3**) The study's stringent case definition of dengue, based on virological confirmation minimised the risk of case misclassification, thereby providing a more precise measurement of dengue incidence and the intervention's impact. (**4**) Our fine-scale aggregation by site, event time, and calendar time allowed for a more granular understanding of *Wolbachia*'s protective effects, revealing dose–response relationships and identifying variations in effectiveness across different locales and time periods.

While this study has its limitations, they do not significantly undermine the integrity of our findings. Firstly, although we were unable to directly measure the percentage of *Wolbachia-*introgression in spillover sites to confirm its actual spatial distribution, the occurrence and impact of this phenomenon have been well-documented in the Yogyakarta RCT, the original Australian releases around Cairns,^15^ as well as around study site W21 (i.e. Mentari Court) in the Malaysian releases.^10^^,^^16^ Secondly, the current recording of dengue infections based on the infected individual's place of residence prevents us from determining whether cases originate from intervention, spillover, or pure control sites. Addressing this would require a resource-intensive prospective study, akin to the Yogyakarta RCT, to obtain a per-protocol effect of *Wolbachia*. Our current intention-to-treat (ITT) analysis likely resulted in conservative effectiveness estimates due to case origin misattribution. Nonetheless, the Yogyakarta study showed that the PE of *Wolbachia* in their per-protocol analyses did not exceed that of their ITT analysis, indicating that despite potential bias in ITT estimates, the observed effectiveness could remain reflective of the true intervention effect.

Overall, these findings showcases the potential for *Wolbachia*-based interventions to extend protective benefits beyond their immediate application areas, supporting their broader adoption in integrated vector management strategies. Future release programs may consider alternative deployment methods to maximise protective coverage of *Wolbachia*. Cluster-based releases, for instance, can create overlapping spillover zones, amplifying *Wolbachia*'s impact by optimising the use of existing resources. Supported by ongoing surveillance, these spatial strategies will enable implementers to adapt *Wolbachia*-interventions in a timely and efficient manner.

## Contributors

JTL and JYC conceptualised the study. AH, CYL, and NWA curated the data. MP and BSLD extracted the environmental covariates. JYC and SB contributed to software. JYC and JTL analysed and validated the data. JYC, JTL, BSLD, and MP drafted the manuscript with contributions in the interpretation of findings from AH, CYL, and NWA. JTL acquired the funding. All authors have read and reviewed the manuscript. JYC, JTL, MP, BSLD, AH, CYL, and NWA accessed and verified the data. All authors had final responsibility to submit for publication.

## Data sharing statements

All code required to replicate the study output is available on GitHub at https://github.com/joyichow/wolb-spillover. The Malaysian dengue incidence, human population and *Wolbachia* frequency data underlying this article is available on Figshare at https://doi.org/10.26188/24314689.v1.

## Declaration of interests

We declare no competing interests.
