# Supplementary material for: Assessing the direct and spillover protective effectiveness of Wolbachia-mediated introgression to combat dengue
Source: eBioMedicine. 2024 Nov 29;110:105456. doi: 10.1016/j.ebiom.2024.105456 (PMC11648191; doi:10.1016/j.ebiom.2024.105456)
Supplement: Supplementary Figures and Tables [file mmc1.docx]

**Supplementary Information for ‘Assessing the direct and spillover protective effectiveness of *Wolbachia*-mediated introgression to combat dengue’**

**Details of the Malaysian operationalised *Wolbachia* trial**

***Wolbachia* strain and pre-release evaluations** The *Wolbachia* strain used in the study by Hoffmann et al. (2024) is *w*AlbB,^1^ introduced into an uninfected local strain of *A. aegypti* from Kuala Lumpur through microinjection techniques. This strain was subsequently transferred to the Institute for Medical Research in Kuala Lumpur from Glasgow University, where it underwent a series of backcrosses to enhance the mosquitoes' fitness and competitive abilities. Wing measurements were conducted periodically for quality control. Additionally, Hoffmann et al. (2024) conducted insecticide bioassays to compare the susceptibility of wild-type and *Wolbachia*-infected *A. aegypti* to common insecticides.^1^ Their results indicated that both groups exhibited similar susceptibility to pyrethroids, organophosphates fenitrothion, and pirimiphos.

**Characteristics of sites and releases** Details of the sites and control sites as well as release and rearing procedures are provided by Nazni et al. (2019) and Hoffmann et al. (2024).^1,2^ The selection of *Wolbachia* release sites was guided by criteria determined by Malaysian health authorities, including dengue incidence, mosquito presence, and perceived barriers, such as roads, that might limit mosquito movement.^3^ 13 of the operational release sites were in Selangor, while five sites were in Kuala Lumpur and two in Putrajaya, bringing to a total of 20 release sites. These sites varied in size, ranging from 19,321 to 276,261 m², where those with high *A. aegypti* and low *A. albopictus* populations were prioritised, determined by an index value of proportions of positive ovitraps placed for a week at sites. 76 control sites in Selangor were identified by the Malaysian Vector Control Department, matched by involving multi-story residential buildings with similar area size, population size and dengue burden. A map of sites is provided by Hoffmann et al. (2024) along with details of release numbers, release frequency and rates of *Wolbachia* invasion.

**Standard Synthetic Control Method**

In the standard (i.e. canonical) synthetic control method (SCM),^4^ synthetic controls are generated specifically and separately for each intervention unit, where each synthetic control is taken as a weighted average of donor (control) units which do not experience intervention. We denote $d=1,2,\ldots,J$ as directly treated sites, and control sites to be $k=I_{0},\ldots,I$. Sites are observed for $t=1,\ldots,t_{j},\ldots,T$ where $t_{j}$ is the adoption time of intervention for some site $j$. $T$ denotes the last time point where dengue incidence rates were observed. Weights $w_{j,i}$ for the control unit $i$ and intervention unit $j$ were estimated by minimising the difference in pre-intervention trends in the outcome variable between the intervention unit $y_{j,{1:t}_{j}-1}$and the donor pool units $y_{i,{1:t}_{j}-1}$:

$\underset{\boldsymbol{w}_{\boldsymbol{j}}}{\mathrm{argmin}} |y_{j}-\sum_{i\in k} w_{j,i}y_{i}|$, {$\sum_{i\in k} w_{j,i}=1,w_{j,i}\geq0$}

where weights are more than or equal to 0 and they sum to 1. Similarly, confidence intervals for the synthetic control weights in standard SCM were obtained by first generating bootstrap samples of dengue incidence rates using the ‘meboot’ package in R, which can create bootstrap timeseries ensembles without assuming stationarity. A total of 10,000 samples were generated, and the SCM was then applied to each bootstrapped timeseries. Bootstrap samples were then used to construct the empirical distribution of the SC weights, from which 95% intervals of protective effectiveness (PE) could be computed.

The counterfactual for some intervention unit is then given as $\sum_{i\in k} \hat{w}_{j,i}y_{i}$and PE in the post-intervention period could be estimated as:

$$PE_{SCM, j}=\sum_{t\in\left\{ t_{j}:T \right\}} \frac{\left( \sum_{i\in k} \hat{w}_{j,i}y_{i,t}-y_{j,t} \right)}{\sum_{i\in k} \hat{w}_{j,i}y_{i,t}} \times100$$

**Supplementary Table S1.** Human population of each directly-intervened and spillover site.^1^

| **Site** | **Population** |
| --- | --- |
| **Directly-intervened sites** | |
| W01 | 4000 |
| W02 | 1560 |
| W03 | 8640 |
| W04 | 8545 |
| W05 | 750 |
| W07 | 10000 |
| W08 | 6000 |
| W09 | 13575 |
| W10 | 9120 |
| W11 | 5760 |
| W15 | 3450 |
| W16 | 4150 |
| W17 | 2450 |
| W18 | 10000 |
| W19 | 5600 |
| W20 | 13955 |
| W21 | 13876 |
| W22 | 11720 |
| W24 | 5632 |
| W26 | 920 |
| W27 | 1960 |
| **Spillover sites** | |
| C43 | 3388 |
| C35 | 3825 |
| C61 | 2500 |
| C39 | 3900 |
| C48 | 1920 |
| C62 | 1000 |
| C25 | 1600 |
| C53 | 3900 |
| C70 | 1150 |
| C03 | 5000 |
| C73 | 1500 |

**Supplementary Table S2.** Distance matrix between directly-intervened sites, calculated using the great circle distance method. The great circle distance represents the shortest path between two points on the surface of a sphere, accounting for the Earth's curvature. The coordinates for each release site were obtained from the data provided by Hoffman et al. (2024).^9^ The matrix values are expressed in metres, indicating the pairwise distances between each site. The diagonal elements represent zero distance, as they compare each site to itself, and are displayed in grey in the associated heatmap to indicate no distance. To prevent redundancy arising from the matrix's inherent symmetry, the upper triangle is also shaded in grey. Distances below 1,500 metres are shaded in red tones, representing relatively closer proximities, while distances above 10,000 metres are uniformly shaded in blue to denote greater separations. Values around 1,500 metres (i.e. designated maximum spillover distance) are highlighted in white to serve as a visual midpoint, distinguishing between near and far proximities.

|  | **W01** | **W02** | **W03** | **W04** | **W05** | **W07** | **W08** | **W09** | **W10** | **W11** | **W15** | **W16** | **W17** | **W18** | **W19** | **W21** | **W22** | **W24** | **W26** | **W27** |
| --- | --- | --- | --- | --- | --- | --- | --- | --- | --- | --- | --- | --- | --- | --- | --- | --- | --- | --- | --- | --- |
| **W01** |  |  |  |  |  |  |  |  |  |  |  |  |  |  |  |  |  |  |  |  |
| **W02** | 10949 |  |  |  |  |  |  |  |  |  |  |  |  |  |  |  |  |  |  |  |
| **W03** | 5340 | 14590 |  |  |  |  |  |  |  |  |  |  |  |  |  |  |  |  |  |  |
| **W04** | 7249 | 15629 | 10924 |  |  |  |  |  |  |  |  |  |  |  |  |  |  |  |  |  |
| **W05** | 5418 | 5581 | 9710 | 10514 |  |  |  |  |  |  |  |  |  |  |  |  |  |  |  |  |
| **W07** | 8799 | 3077 | 13187 | 12685 | 3494 |  |  |  |  |  |  |  |  |  |  |  |  |  |  |  |
| **W08** | 11069 | 13021 | 8750 | 18259 | 11271 | 13574 |  |  |  |  |  |  |  |  |  |  |  |  |  |  |
| **W09** | 12943 | 23360 | 13192 | 8590 | 17848 | 20650 | 21932 |  |  |  |  |  |  |  |  |  |  |  |  |  |
| **W10** | 14046 | 24012 | 14943 | 8633 | 18615 | 21174 | 23621 | 2220 |  |  |  |  |  |  |  |  |  |  |  |  |
| **W11** | 10196 | 18850 | 13059 | 3251 | 13764 | 15876 | 20986 | 6483 | 5883 |  |  |  |  |  |  |  |  |  |  |  |
| **W15** | 17285 | 28187 | 14420 | 16287 | 22702 | 26051 | 22184 | 9126 | 10974 | 15182 |  |  |  |  |  |  |  |  |  |  |
| **W16** | 22566 | 28403 | 26032 | 15373 | 24578 | 25362 | 33621 | 16158 | 14020 | 12990 | 24823 |  |  |  |  |  |  |  |  |  |
| **W17** | 22691 | 28477 | 26174 | 15504 | 24675 | 25439 | 33748 | 16327 | 14190 | 13136 | 24994 | 171 |  |  |  |  |  |  |  |  |
| **W18** | 5550 | 12450 | 10372 | 3218 | 7516 | 9479 | 16485 | 11601 | 11820 | 6402 | 18591 | 17414 | 17526 |  |  |  |  |  |  |  |
| **W19** | 5404 | 14481 | 256 | 11117 | 9658 | 13126 | 8494 | 13448 | 15197 | 13282 | 14625 | 26250 | 26392 | 10507 |  |  |  |  |  |  |
| **W21** | 3300 | 12118 | 7985 | 4126 | 6693 | 9396 | 14361 | 11261 | 11926 | 7280 | 17254 | 19306 | 19428 | 2389 | 8118 |  |  |  |  |  |
| **W22** | 12906 | 1964 | 16413 | 17499 | 7545 | 4831 | 14115 | 25308 | 25931 | 20702 | 30125 | 29883 | 29951 | 14301 | 16293 | 14057 |  |  |  |  |
| **W24** | 12420 | 1532 | 16122 | 16804 | 7019 | 4119 | 14276 | 24693 | 25273 | 19994 | 29681 | 29080 | 29147 | 13598 | 16013 | 13433 | 810 |  |  |  |
| **W26** | 12126 | 1206 | 15635 | 16815 | 6777 | 4193 | 13527 | 24566 | 25214 | 20029 | 29339 | 29405 | 29476 | 13627 | 15516 | 13323 | 787 | 790 |  |  |
| **W27** | 12164 | 1301 | 15610 | 16930 | 6836 | 4336 | 13388 | 24646 | 25310 | 20148 | 29358 | 29581 | 29652 | 13748 | 15488 | 13411 | 809 | 982 | 214 |  |

**Supplementary Table S3.** Weights of environmental covariates for each site using Model 2, where the average of each covariate across the pre-intervention period was utilised ($M_{avg}$), along with their corresponding pre-intervention root-mean-square errors (RMSEs). The weights assigned to each covariate indicate their relative importance in the synthetic control model prediction across different sites, while the RMSE values assess the accuracy of the synthetic control model predictions. RMSEs highlighted in red indicate that Model 2’s prediction for the site had a higher RMSE than the baseline model (i.e. synthetic control without covariates), which was used in subsequent analyses.

| **Site** |  | **Environmental covariate** | | | | | | | | |
| --- | --- | --- | --- | --- | --- | --- | --- | --- | --- | --- |
|  | **RMSE** | **Temperature**  **(℃)** | **2m temperature**  **(℃)** | **Total precipitation**  **(mm)** | **Relative humidity**  **(%)** | **Fraction of cloud cover**  **(0-1)** | **Low vegetation cover**  **(0-1)** | **High vegetation cover**  **(0-1)** | **Leaf area index, low vegetation (m²/m²)** | **Leaf area index, high vegetation (m²/m²)** |
| **Directly-intervened sites** | | | | | | | | | | |
| W01 | 90·93 | 1·8E-08 | 1·2E-04 | 1·8E-08 | 1·8E-08 | 1·8E-08 | 1·8E-08 | 4·9E-01 | 2·2E-07 | 5·1E-01 |
| W02 | 97·83 | 2·6E-08 | 2·6E-08 | 2·6E-08 | 2·6E-08 | 1·4E-04 | 2·6E-08 | 5·3E-01 | 1·0E-04 | 4·7E-01 |
| W03 | 23·45 | 2·5E-09 | 2·5E-01 | 2·5E-01 | 2·5E-01 | 2·5E-09 | 2·5E-01 | 2·5E-09 | 2·5E-09 | 7·5E-07 |
| W04 | 19·38 | 3·3E-09 | 3·3E-01 | 3·3E-09 | 3·3E-01 | 3·3E-01 | 9·2E-07 | 3·3E-09 | 3·3E-09 | 3·3E-09 |
| W05 | 522·73 | 4·7E-02 | 4·8E-01 | 8·8E-09 | 4·7E-01 | 8·8E-09 | 8·8E-09 | 3·7E-07 | 8·8E-09 | 8·8E-09 |
| W07 | 44·76 | 1·0E-08 | 1·0E-08 | 1·0E-08 | 1·0E+00 | 2·0E-04 | 1·0E-08 | 1·2E-05 | 1·0E-08 | 1·0E-08 |
| W08 | 32·38 | 4·3E-09 | 4·3E-09 | 1·1E-07 | 4·3E-09 | 4·0E-01 | 4·3E-09 | 4·1E-01 | 4·3E-09 | 1·9E-01 |
| W09 | 15·66 | 3·3E-09 | 3·3E-09 | 3·3E-09 | 3·3E-09 | 3·3E-01 | 3·3E-09 | 3·3E-01 | 3·3E-01 | 1·7E-07 |
| W10 | 12·20 | 5·0E-09 | 5·0E-01 | 5·0E-09 | 1·0E-04 | 1·6E-03 | 5·0E-09 | 5·0E-01 | 5·0E-09 | 5·0E-09 |
| W11 | 15·78 | 3·3E-01 | 4·2E-09 | 3·3E-01 | 4·2E-09 | 1·7E-08 | 3·4E-01 | 1·7E-06 | 4·2E-09 | 4·2E-09 |
| W15 | 31·53 | 3·7E-09 | 3·3E-01 | 3·3E-01 | 3·3E-01 | 3·7E-04 | 1·6E-03 | 3·7E-09 | 3·7E-09 | 5·3E-08 |
| W16 | 35·64 | 1·6E-08 | 1·6E-08 | 1·7E-05 | 1·6E-08 | 1·0E+00 | 1·6E-08 | 1·6E-08 | 6·5E-04 | 4·6E-06 |
| W17 | 21·85 | 1·0E-08 | 1·0E-08 | 1·3E-06 | 1·0E+00 | 1·0E-08 | 1·0E-08 | 1·0E-08 | 1·0E-08 | 1·0E-08 |
| W18 | 35·43 | 3·3E-09 | 2·9E-07 | 9·1E-07 | 3·3E-09 | 3·3E-01 | 3·3E-01 | 3·3E-09 | 3·3E-09 | 3·3E-01 |
| W19 | 22·91 | 8·3E-09 | 3·0E-04 | 4·9E-01 | 5·1E-01 | 9·3E-06 | 8·3E-09 | 8·3E-09 | 8·3E-09 | 7·4E-07 |
| W21 | 35·42 | 8·1E-07 | 9·6E-01 | 2·3E-02 | 6·5E-03 | 8·1E-07 | 8·1E-07 | 8·1E-07 | 8·1E-07 | 5·9E-03 |
| W22 | 49·44 | 4·9E-01 | 6·6E-04 | 6·6E-04 | 4·9E-01 | 6·6E-04 | 6·6E-04 | 9·9E-03 | 8·5E-03 | 6·6E-04 |
| W24 | 88·81 | 1·5E-07 | 5·0E-09 | 5·0E-09 | 5·0E-09 | 5·0E-01 | 5·0E-09 | 5·0E-01 | 1·2E-06 | 5·0E-09 |
| W26 | 58·13 | 2·9E-08 | 1·9E-05 | 2·9E-08 | 2·9E-08 | 1·0E+00 | 3·8E-06 | 2·9E-08 | 2·9E-08 | 2·9E-08 |
| W27 | 135·11 | 6·2E-09 | 6·2E-09 | 6·2E-09 | 6·2E-09 | 1·5E-05 | 6·2E-09 | 5·0E-01 | 1·9E-05 | 5·0E-01 |
| **Spillover sites** | | | | | | | | | | |
| C43 | 52·69 | 3·4E-07 | 3·4E-07 | 4·9E-03 | 3·4E-07 | 6·4E-01 | 3·4E-07 | 3·4E-07 | 2·4E-02 | 3·3E-01 |
| C35 | 45·99 | 5·0E-09 | 5·0E-09 | 5·0E-01 | 5·0E-09 | 1·7E-03 | 5·0E-09 | 5·0E-09 | 5·0E-01 | 9·6E-05 |
| C61 | 50·66 | 3·3E-09 | 3·3E-01 | 3·3E-09 | 3·3E-01 | 3·3E-09 | 3·3E-01 | 8·0E-07 | 3·3E-09 | 3·3E-09 |
| C39 | 88·47 | 5·0E-09 | 5·0E-09 | 5·0E-01 | 5·0E-09 | 5·0E-01 | 5·0E-09 | 4·4E-06 | 2·2E-03 | 9·0E-05 |
| C48 | 124·12 | 5·0E-09 | 5·0E-09 | 5·0E-09 | 5·9E-04 | 5·0E-01 | 1·5E-05 | 5·0E-09 | 5·0E-09 | 5·0E-01 |
| C62 | 122·38 | 5·5E-01 | 3·1E-08 | 3·1E-08 | 3·1E-08 | 3·1E-08 | 4·5E-01 | 2·8E-07 | 1·2E-06 | 3·1E-08 |
| C25 | 55·50 | 2·5E-01 | 2·5E-09 | 2·5E-09 | 2·5E-09 | 2·5E-09 | 2·5E-01 | 2·5E-01 | 2·5E-09 | 2·5E-01 |
| C53 | 97·16 | 1·3E-04 | 3·3E-09 | 3·3E-09 | 3·3E-09 | 3·3E-09 | 3·3E-01 | 3·3E-01 | 3·3E-01 | 3·3E-09 |
| C70 | 29·08 | 1·0E-08 | 3·0E-05 | 1·0E-08 | 1·5E-01 | 2·2E-05 | 7·3E-02 | 1·0E-08 | 7·7E-01 | 1·3E-08 |
| C03 | 118·38 | 5·0E-09 | 5·0E-01 | 5·0E-09 | 5·0E-01 | 2·4E-06 | 2·9E-07 | 3·9E-08 | 5·0E-09 | 5·0E-09 |
| C73 | 38·04 | 2·0E-08 | 5·6E-01 | 4·4E-01 | 2·0E-06 | 2·0E-08 | 1·5E-06 | 2·0E-08 | 2·0E-08 | 4·4E-07 |

**Supplementary Table S4.** Weights for each donor (control) site in the construction of directly-intervened sites’ synthetic controls in base synthetic control model (i.e. partial interference SCM without covariates). Weights assigned to each donor site represent their relative contributions to the construction of the synthetic control site.

| **Donor site** | W01 | W02 | W03 | W04 | W05 | W07 | W08 | W09 | W10 | W11 | W15 | W16 | W17 | W18 | W19 | W21 | W22 | W24 | W26 | W27 |
| --- | --- | --- | --- | --- | --- | --- | --- | --- | --- | --- | --- | --- | --- | --- | --- | --- | --- | --- | --- | --- |
| C01 | 0·1702 | 0·0000 | 0·0074 | 0·0000 | 0·0000 | 0·0000 | 0·0000 | 0·0000 | 0·0000 | 0·0000 | 0·0000 | 0·0000 | 0·0000 | 0·0000 | 0·0156 | 0·0000 | 0·0000 | 0·0000 | 0·0000 | 0·0000 |
| C02 | 0·0000 | 0·0000 | 0·0200 | 0·0000 | 0·0000 | 0·0000 | 0·0000 | 0·0000 | 0·0000 | 0·0470 | 0·0000 | 0·0000 | 0·0000 | 0·0000 | 0·0000 | 0·0000 | 0·0000 | 0·0000 | 0·0000 | 0·0000 |
| C04 | 0·0000 | 0·0000 | 0·0000 | 0·0000 | 0·0000 | 0·0275 | 0·0000 | 0·0000 | 0·0000 | 0·0000 | 0·0222 | 0·0000 | 0·0000 | 0·0215 | 0·0000 | 0·1275 | 0·0000 | 0·0000 | 0·0000 | 0·1914 |
| C05 | 0·0000 | 0·0000 | 0·1085 | 0·0000 | 0·0000 | 0·0000 | 0·0000 | 0·1573 | 0·0403 | 0·0000 | 0·0000 | 0·0000 | 0·0000 | 0·0000 | 0·0000 | 0·0000 | 0·0000 | 0·0000 | 0·0000 | 0·0000 |
| C06 | 0·0847 | 0·0000 | 0·0000 | 0·0000 | 0·0000 | 0·0681 | 0·0341 | 0·0000 | 0·0347 | 0·0290 | 0·0000 | 0·0000 | 0·0000 | 0·0146 | 0·0000 | 0·0000 | 0·0000 | 0·0000 | 0·0000 | 0·0000 |
| C07 | 0·0000 | 0·0000 | 0·1383 | 0·0000 | 0·0000 | 0·0377 | 0·0000 | 0·2838 | 0·1013 | 0·0798 | 0·1492 | 0·1650 | 0·1467 | 0·0000 | 0·0314 | 0·0000 | 0·0000 | 0·0000 | 0·3303 | 0·0000 |
| C08 | 0·0000 | 0·0000 | 0·0047 | 0·0153 | 0·0000 | 0·0833 | 0·1387 | 0·0000 | 0·0182 | 0·1564 | 0·1297 | 0·0000 | 0·0389 | 0·1935 | 0·0020 | 0·0004 | 0·0000 | 0·0000 | 0·0000 | 0·0000 |
| C09 | 0·0000 | 0·0000 | 0·0000 | 0·0000 | 0·0000 | 0·0079 | 0·0000 | 0·0000 | 0·0000 | 0·0000 | 0·0000 | 0·0000 | 0·0000 | 0·0000 | 0·0000 | 0·0886 | 0·0000 | 0·0000 | 0·0000 | 0·0000 |
| C10 | 0·0000 | 0·0000 | 0·1371 | 0·1530 | 0·0000 | 0·0000 | 0·0000 | 0·1308 | 0·1346 | 0·1911 | 0·0000 | 0·0477 | 0·1968 | 0·0000 | 0·2239 | 0·0000 | 0·0000 | 0·0000 | 0·0000 | 0·0000 |
| C11 | 0·0000 | 0·0813 | 0·0000 | 0·0000 | 0·0000 | 0·0000 | 0·0000 | 0·0000 | 0·0000 | 0·0000 | 0·0000 | 0·0000 | 0·0000 | 0·0000 | 0·0000 | 0·0000 | 0·0000 | 0·0562 | 0·0142 | 0·0000 |
| C12 | 0·0000 | 0·0000 | 0·0159 | 0·0384 | 0·0000 | 0·0000 | 0·0000 | 0·0657 | 0·0000 | 0·0000 | 0·0000 | 0·1410 | 0·0253 | 0·0000 | 0·0000 | 0·0000 | 0·0000 | 0·0000 | 0·0000 | 0·0000 |
| C13 | 0·0000 | 0·0000 | 0·0000 | 0·0000 | 0·0000 | 0·0000 | 0·0068 | 0·0000 | 0·0000 | 0·0000 | 0·0000 | 0·0000 | 0·0000 | 0·0000 | 0·0000 | 0·0260 | 0·0161 | 0·0000 | 0·0000 | 0·0000 |
| C14 | 0·0000 | 0·0000 | 0·1006 | 0·1908 | 0·0000 | 0·0000 | 0·0000 | 0·0139 | 0·1287 | 0·0658 | 0·0639 | 0·0000 | 0·0647 | 0·0000 | 0·0167 | 0·0000 | 0·0000 | 0·0000 | 0·0000 | 0·0000 |
| C15 | 0·0000 | 0·0000 | 0·0063 | 0·0343 | 0·0000 | 0·0467 | 0·0137 | 0·0123 | 0·0065 | 0·0038 | 0·0000 | 0·0000 | 0·0000 | 0·0000 | 0·0079 | 0·0584 | 0·0436 | 0·0000 | 0·0000 | 0·0000 |
| C16 | 0·0000 | 0·0000 | 0·0000 | 0·0645 | 0·0000 | 0·0014 | 0·0000 | 0·0000 | 0·0000 | 0·0186 | 0·0000 | 0·0000 | 0·0000 | 0·0000 | 0·0296 | 0·1235 | 0·0000 | 0·0000 | 0·0000 | 0·0000 |
| C17 | 0·0000 | 0·0000 | 0·0000 | 0·0000 | 0·0000 | 0·0299 | 0·0450 | 0·0000 | 0·0000 | 0·0018 | 0·0000 | 0·0000 | 0·0000 | 0·0181 | 0·0124 | 0·0043 | 0·0000 | 0·0000 | 0·0000 | 0·0000 |
| C19 | 0·0000 | 0·0000 | 0·0000 | 0·0000 | 0·0000 | 0·0000 | 0·1689 | 0·0000 | 0·0000 | 0·0000 | 0·0000 | 0·0000 | 0·0000 | 0·0000 | 0·0603 | 0·0000 | 0·0000 | 0·0000 | 0·0000 | 0·0000 |
| C20 | 0·0000 | 0·0000 | 0·0000 | 0·0000 | 0·0000 | 0·0022 | 0·0000 | 0·0000 | 0·0000 | 0·0000 | 0·0000 | 0·0000 | 0·0000 | 0·0676 | 0·0000 | 0·0000 | 0·0000 | 0·0000 | 0·0000 | 0·0000 |
| C21 | 0·0000 | 0·0121 | 0·0000 | 0·0321 | 0·0000 | 0·0000 | 0·0161 | 0·0000 | 0·0000 | 0·0000 | 0·0000 | 0·0000 | 0·0000 | 0·0000 | 0·0000 | 0·0036 | 0·0429 | 0·2617 | 0·0000 | 0·0000 |
| C22 | 0·0000 | 0·0000 | 0·0543 | 0·0000 | 0·0000 | 0·0000 | 0·0000 | 0·0917 | 0·0437 | 0·0829 | 0·0000 | 0·0000 | 0·0000 | 0·0000 | 0·0470 | 0·0000 | 0·0000 | 0·0000 | 0·0000 | 0·0000 |
| C23 | 0·0000 | 0·0388 | 0·0000 | 0·0000 | 0·0000 | 0·0284 | 0·0710 | 0·0000 | 0·0000 | 0·0000 | 0·0000 | 0·0000 | 0·0000 | 0·0000 | 0·0022 | 0·0000 | 0·0000 | 0·0000 | 0·0000 | 0·0000 |
| C24 | 0·0000 | 0·0000 | 0·0000 | 0·0000 | 0·0000 | 0·0000 | 0·0000 | 0·1001 | 0·0146 | 0·0376 | 0·0644 | 0·0891 | 0·0752 | 0·0069 | 0·0133 | 0·0000 | 0·0000 | 0·0000 | 0·0000 | 0·0000 |
| C26 | 0·0000 | 0·0000 | 0·0000 | 0·0000 | 0·0000 | 0·0000 | 0·0000 | 0·0000 | 0·0000 | 0·0000 | 0·0000 | 0·0000 | 0·0000 | 0·0000 | 0·0000 | 0·0470 | 0·0920 | 0·0124 | 0·0000 | 0·0000 |
| C27 | 0·0000 | 0·0000 | 0·0034 | 0·0227 | 0·0000 | 0·0000 | 0·0639 | 0·0000 | 0·0000 | 0·0000 | 0·0231 | 0·0000 | 0·0071 | 0·0488 | 0·0000 | 0·0000 | 0·0116 | 0·0000 | 0·0000 | 0·0000 |
| C28 | 0·0000 | 0·0000 | 0·0000 | 0·0333 | 0·0000 | 0·0000 | 0·0000 | 0·0000 | 0·0000 | 0·0407 | 0·0000 | 0·0650 | 0·0000 | 0·1487 | 0·0000 | 0·0719 | 0·0378 | 0·0000 | 0·0000 | 0·0000 |
| C29 | 0·0441 | 0·0000 | 0·0000 | 0·0085 | 0·0000 | 0·0000 | 0·0000 | 0·0098 | 0·0000 | 0·0062 | 0·0233 | 0·0000 | 0·0000 | 0·0000 | 0·0000 | 0·0000 | 0·0735 | 0·0000 | 0·0000 | 0·1814 |
| C30 | 0·0000 | 0·0000 | 0·0000 | 0·0975 | 0·0000 | 0·0000 | 0·0000 | 0·0000 | 0·2348 | 0·0113 | 0·0000 | 0·0075 | 0·1394 | 0·0584 | 0·0000 | 0·0159 | 0·0000 | 0·0000 | 0·0000 | 0·0000 |
| C31 | 0·0000 | 0·0000 | 0·0000 | 0·0000 | 0·0000 | 0·0301 | 0·0000 | 0·0000 | 0·0000 | 0·0000 | 0·0000 | 0·0000 | 0·0000 | 0·0000 | 0·0179 | 0·0087 | 0·0000 | 0·0027 | 0·0000 | 0·0000 |
| C32 | 0·0000 | 0·0000 | 0·0128 | 0·0000 | 0·0000 | 0·0000 | 0·0000 | 0·0000 | 0·0000 | 0·0000 | 0·0000 | 0·0000 | 0·0000 | 0·0000 | 0·0000 | 0·0159 | 0·0000 | 0·0000 | 0·0000 | 0·0000 |
| C33 | 0·0560 | 0·1093 | 0·0000 | 0·0000 | 0·0000 | 0·0282 | 0·0256 | 0·0000 | 0·0000 | 0·0000 | 0·0176 | 0·0000 | 0·0000 | 0·0274 | 0·0000 | 0·0176 | 0·0000 | 0·1413 | 0·0145 | 0·0000 |
| C34 | 0·0113 | 0·0000 | 0·0047 | 0·0016 | 0·0000 | 0·0000 | 0·0000 | 0·0000 | 0·0010 | 0·0000 | 0·0028 | 0·0000 | 0·0000 | 0·0000 | 0·0000 | 0·0000 | 0·0662 | 0·0000 | 0·0000 | 0·0000 |
| C36 | 0·3850 | 0·0000 | 0·0000 | 0·0000 | 0·0000 | 0·0000 | 0·0000 | 0·0000 | 0·0000 | 0·0000 | 0·0000 | 0·0214 | 0·0066 | 0·1770 | 0·0352 | 0·0101 | 0·0000 | 0·0000 | 0·0114 | 0·0000 |
| C37 | 0·0000 | 0·0000 | 0·0000 | 0·0000 | 0·0000 | 0·0000 | 0·0000 | 0·0000 | 0·0000 | 0·0000 | 0·1325 | 0·0000 | 0·0000 | 0·0000 | 0·0000 | 0·0000 | 0·0000 | 0·0000 | 0·0000 | 0·0000 |
| C38 | 0·0000 | 0·0430 | 0·0000 | 0·0084 | 0·1910 | 0·0000 | 0·0139 | 0·0031 | 0·0000 | 0·0000 | 0·0008 | 0·0000 | 0·0000 | 0·0080 | 0·0000 | 0·0078 | 0·0185 | 0·0370 | 0·0000 | 0·1224 |
| C40 | 0·0000 | 0·0000 | 0·0000 | 0·0000 | 0·0000 | 0·0178 | 0·0000 | 0·0018 | 0·0000 | 0·0000 | 0·0208 | 0·0182 | 0·0025 | 0·0000 | 0·0000 | 0·0000 | 0·0000 | 0·0000 | 0·0883 | 0·0000 |
| C41 | 0·0000 | 0·0000 | 0·1333 | 0·1027 | 0·0000 | 0·0000 | 0·0000 | 0·0000 | 0·0332 | 0·0000 | 0·0000 | 0·0000 | 0·0000 | 0·0000 | 0·0000 | 0·0974 | 0·0921 | 0·0000 | 0·3042 | 0·0000 |
| C42 | 0·0000 | 0·0000 | 0·0000 | 0·0000 | 0·0000 | 0·0000 | 0·0000 | 0·0000 | 0·0000 | 0·0000 | 0·0000 | 0·0000 | 0·0000 | 0·0000 | 0·0000 | 0·0000 | 0·0000 | 0·0000 | 0·0000 | 0·0000 |
| C44 | 0·0000 | 0·0000 | 0·0000 | 0·0000 | 0·0000 | 0·0000 | 0·0000 | 0·0000 | 0·0000 | 0·0000 | 0·0000 | 0·0000 | 0·0000 | 0·0000 | 0·0047 | 0·0000 | 0·0000 | 0·0000 | 0·0000 | 0·0000 |
| C45 | 0·0000 | 0·0000 | 0·1193 | 0·0000 | 0·0000 | 0·0000 | 0·0000 | 0·0824 | 0·1010 | 0·1115 | 0·0506 | 0·1960 | 0·1445 | 0·0000 | 0·2144 | 0·0000 | 0·0000 | 0·0000 | 0·0000 | 0·0000 |
| C46 | 0·0000 | 0·0000 | 0·0481 | 0·0000 | 0·0000 | 0·0000 | 0·0000 | 0·0000 | 0·0000 | 0·0000 | 0·0000 | 0·0000 | 0·0000 | 0·0000 | 0·0000 | 0·0653 | 0·0271 | 0·0000 | 0·0000 | 0·0000 |
| C47 | 0·0000 | 0·0000 | 0·0000 | 0·0827 | 0·0000 | 0·0000 | 0·1386 | 0·0000 | 0·1047 | 0·0837 | 0·0000 | 0·0000 | 0·1126 | 0·0000 | 0·1267 | 0·0000 | 0·0000 | 0·0000 | 0·0000 | 0·0000 |
| C49 | 0·0000 | 0·0000 | 0·0235 | 0·0000 | 0·0000 | 0·0000 | 0·0000 | 0·0000 | 0·0000 | 0·0000 | 0·0000 | 0·0000 | 0·0000 | 0·0000 | 0·0017 | 0·0000 | 0·0000 | 0·0000 | 0·0000 | 0·0000 |
| C50 | 0·0061 | 0·0440 | 0·0000 | 0·0000 | 0·1954 | 0·0000 | 0·0103 | 0·0000 | 0·0000 | 0·0000 | 0·0000 | 0·0000 | 0·0000 | 0·0000 | 0·0000 | 0·0030 | 0·0000 | 0·0339 | 0·0000 | 0·0991 |
| C51 | 0·0000 | 0·0000 | 0·0000 | 0·0000 | 0·0000 | 0·0000 | 0·0000 | 0·0000 | 0·0000 | 0·0000 | 0·0000 | 0·0000 | 0·0000 | 0·0566 | 0·0000 | 0·0000 | 0·0186 | 0·0000 | 0·0000 | 0·1885 |
| C52 | 0·0000 | 0·0177 | 0·0000 | 0·0000 | 0·5317 | 0·0000 | 0·0192 | 0·0000 | 0·0000 | 0·0000 | 0·0000 | 0·0000 | 0·0000 | 0·0190 | 0·0000 | 0·0388 | 0·0125 | 0·0837 | 0·0000 | 0·0000 |
| C54 | 0·0000 | 0·0000 | 0·0000 | 0·0000 | 0·0000 | 0·0000 | 0·0000 | 0·0000 | 0·0000 | 0·0000 | 0·0000 | 0·0000 | 0·0000 | 0·0063 | 0·0000 | 0·0000 | 0·0359 | 0·2888 | 0·0000 | 0·0937 |
| C55 | 0·0000 | 0·0000 | 0·0000 | 0·0000 | 0·0000 | 0·0584 | 0·0000 | 0·0000 | 0·0000 | 0·0000 | 0·0000 | 0·0000 | 0·0000 | 0·0013 | 0·0000 | 0·0000 | 0·0000 | 0·0000 | 0·0000 | 0·0000 |
| C56 | 0·0000 | 0·2473 | 0·0117 | 0·0000 | 0·0000 | 0·0072 | 0·0000 | 0·0299 | 0·0000 | 0·0000 | 0·0011 | 0·1686 | 0·0140 | 0·0000 | 0·0065 | 0·0000 | 0·0256 | 0·0000 | 0·0618 | 0·0000 |
| C57 | 0·0000 | 0·0336 | 0·0000 | 0·0000 | 0·0000 | 0·1767 | 0·0000 | 0·0111 | 0·0028 | 0·0000 | 0·0426 | 0·0000 | 0·0000 | 0·0384 | 0·0321 | 0·0000 | 0·0257 | 0·0000 | 0·0103 | 0·0000 |
| C58 | 0·0158 | 0·2401 | 0·0000 | 0·0000 | 0·0000 | 0·0000 | 0·0406 | 0·0000 | 0·0000 | 0·0006 | 0·0827 | 0·0167 | 0·0000 | 0·0018 | 0·0000 | 0·0094 | 0·0191 | 0·0000 | 0·0172 | 0·0000 |
| C59 | 0·0000 | 0·0000 | 0·0000 | 0·1108 | 0·0000 | 0·0000 | 0·1403 | 0·0000 | 0·0000 | 0·0000 | 0·1156 | 0·0000 | 0·0000 | 0·0000 | 0·0000 | 0·0639 | 0·0000 | 0·0000 | 0·0000 | 0·0000 |
| C60 | 0·0000 | 0·0000 | 0·0000 | 0·0000 | 0·0000 | 0·0101 | 0·0000 | 0·0000 | 0·0000 | 0·0000 | 0·0000 | 0·0000 | 0·0000 | 0·0000 | 0·0155 | 0·0253 | 0·0000 | 0·0000 | 0·0000 | 0·0000 |
| C63 | 0·0000 | 0·0000 | 0·0000 | 0·0000 | 0·0000 | 0·0000 | 0·0210 | 0·0000 | 0·0000 | 0·0000 | 0·0000 | 0·0000 | 0·0000 | 0·0145 | 0·0000 | 0·0120 | 0·0512 | 0·0000 | 0·0000 | 0·0255 |
| C65 | 0·0000 | 0·0000 | 0·0000 | 0·0000 | 0·0000 | 0·0000 | 0·0000 | 0·0000 | 0·0000 | 0·0000 | 0·0000 | 0·0000 | 0·0000 | 0·0000 | 0·0000 | 0·0000 | 0·0064 | 0·0000 | 0·0000 | 0·0000 |
| C66 | 0·0000 | 0·0000 | 0·0000 | 0·0000 | 0·0000 | 0·0000 | 0·0058 | 0·0000 | 0·0000 | 0·0000 | 0·0000 | 0·0000 | 0·0000 | 0·0536 | 0·0000 | 0·0440 | 0·0222 | 0·0396 | 0·0000 | 0·0000 |
| C67 | 0·0000 | 0·0341 | 0·0064 | 0·0000 | 0·0000 | 0·0716 | 0·0000 | 0·0063 | 0·0000 | 0·0000 | 0·0000 | 0·0637 | 0·0257 | 0·0000 | 0·0111 | 0·0000 | 0·0000 | 0·0000 | 0·0296 | 0·0000 |
| C68 | 0·0000 | 0·0000 | 0·0000 | 0·0000 | 0·0000 | 0·0202 | 0·0000 | 0·0000 | 0·0000 | 0·0000 | 0·0000 | 0·0000 | 0·0000 | 0·0000 | 0·0000 | 0·0137 | 0·0000 | 0·0000 | 0·0000 | 0·0000 |
| C69 | 0·1069 | 0·0000 | 0·0000 | 0·0000 | 0·0000 | 0·0150 | 0·0000 | 0·0000 | 0·0000 | 0·0000 | 0·0000 | 0·0000 | 0·0000 | 0·0000 | 0·0000 | 0·0000 | 0·0000 | 0·0000 | 0·0000 | 0·0707 |
| C71 | 0·1199 | 0·0141 | 0·0000 | 0·0000 | 0·0367 | 0·0199 | 0·0000 | 0·0000 | 0·0000 | 0·0000 | 0·0000 | 0·0000 | 0·0000 | 0·0000 | 0·0000 | 0·0000 | 0·0296 | 0·0427 | 0·0000 | 0·0273 |
| C72 | 0·0000 | 0·0000 | 0·0000 | 0·0000 | 0·0000 | 0·0847 | 0·0000 | 0·0000 | 0·0000 | 0·0000 | 0·0571 | 0·0000 | 0·0000 | 0·0000 | 0·0000 | 0·0000 | 0·0000 | 0·0000 | 0·0000 | 0·0000 |
| C74 | 0·0000 | 0·0000 | 0·0000 | 0·0000 | 0·0000 | 0·0000 | 0·0000 | 0·0000 | 0·0000 | 0·0000 | 0·0000 | 0·0000 | 0·0000 | 0·0000 | 0·0000 | 0·0000 | 0·0000 | 0·0000 | 0·1067 | 0·0000 |
| C76 | 0·0000 | 0·0000 | 0·0000 | 0·0000 | 0·0000 | 0·0050 | 0·0000 | 0·0000 | 0·0000 | 0·0000 | 0·0000 | 0·0000 | 0·0000 | 0·0000 | 0·0000 | 0·0000 | 0·0000 | 0·0000 | 0·0000 | 0·0000 |
| C77 | 0·0000 | 0·0000 | 0·0231 | 0·0000 | 0·0000 | 0·0003 | 0·0000 | 0·0000 | 0·0000 | 0·0000 | 0·0000 | 0·0000 | 0·0000 | 0·0000 | 0·0000 | 0·0000 | 0·1073 | 0·0000 | 0·0000 | 0·0000 |
| C78 | 0·0000 | 0·0845 | 0·0000 | 0·0034 | 0·0452 | 0·0000 | 0·0265 | 0·0000 | 0·0000 | 0·0000 | 0·0000 | 0·0000 | 0·0000 | 0·0179 | 0·0000 | 0·0000 | 0·1243 | 0·0000 | 0·0115 | 0·0000 |
| C79 | 0·0000 | 0·0000 | 0·0204 | 0·0000 | 0·0000 | 0·1219 | 0·0000 | 0·0000 | 0·0000 | 0·0320 | 0·0000 | 0·0000 | 0·0000 | 0·0000 | 0·0721 | 0·0000 | 0·0000 | 0·0000 | 0·0000 | 0·0000 |

**Supplementary Table S5.** Weights for each donor (control) site in the construction of spillover sites’ synthetic controls in the base synthetic control model (i.e. partial interference SCM without covariates).

| **Donor site** | C43 | C35 | C61 | C39 | C48 | C62 | C25 | C53 | C70 | C03 | C73 |
| --- | --- | --- | --- | --- | --- | --- | --- | --- | --- | --- | --- |
| C01 | 0·0000 | 0·0000 | 0·0224 | 0·0000 | 0·0000 | 0·0000 | 0·1795 | 0·0000 | 0·0000 | 0·0000 | 0·0000 |
| C02 | 0·0000 | 0·4393 | 0·0000 | 0·0000 | 0·0000 | 0·0000 | 0·0000 | 0·0000 | 0·0000 | 0·0000 | 0·0000 |
| C04 | 0·0005 | 0·0052 | 0·0000 | 0·1130 | 0·0000 | 0·0000 | 0·0000 | 0·0221 | 0·0000 | 0·0000 | 0·0000 |
| C05 | 0·0000 | 0·0000 | 0·0000 | 0·0000 | 0·0000 | 0·0000 | 0·0000 | 0·0000 | 0·0454 | 0·0000 | 0·0000 |
| C06 | 0·0000 | 0·0000 | 0·2474 | 0·0000 | 0·0000 | 0·0000 | 0·1103 | 0·0000 | 0·0000 | 0·0000 | 0·0000 |
| C07 | 0·0000 | 0·0000 | 0·0000 | 0·0000 | 0·0000 | 0·0000 | 0·0000 | 0·0000 | 0·0385 | 0·0000 | 0·1053 |
| C08 | 0·0000 | 0·0000 | 0·0000 | 0·0000 | 0·0000 | 0·0000 | 0·0000 | 0·0000 | 0·0000 | 0·0000 | 0·0000 |
| C09 | 0·0000 | 0·0000 | 0·0000 | 0·0000 | 0·0000 | 0·0000 | 0·0314 | 0·0000 | 0·0000 | 0·0000 | 0·0000 |
| C10 | 0·0000 | 0·0000 | 0·0000 | 0·0000 | 0·0000 | 0·0000 | 0·0000 | 0·0000 | 0·0000 | 0·0000 | 0·0000 |
| C11 | 0·0000 | 0·0000 | 0·0000 | 0·0609 | 0·0000 | 0·0000 | 0·0000 | 0·0000 | 0·0016 | 0·0000 | 0·0000 |
| C12 | 0·0000 | 0·0780 | 0·0000 | 0·0000 | 0·0000 | 0·0000 | 0·0000 | 0·0000 | 0·0000 | 0·0000 | 0·0000 |
| C13 | 0·0710 | 0·0093 | 0·0919 | 0·0000 | 0·0000 | 0·0000 | 0·0000 | 0·0564 | 0·0000 | 0·0000 | 0·0339 |
| C14 | 0·0000 | 0·0000 | 0·0000 | 0·0000 | 0·0000 | 0·0000 | 0·0000 | 0·0000 | 0·0288 | 0·0000 | 0·0817 |
| C15 | 0·0000 | 0·0358 | 0·0000 | 0·0000 | 0·0000 | 0·0000 | 0·0000 | 0·0000 | 0·0000 | 0·0000 | 0·0000 |
| C16 | 0·0000 | 0·0000 | 0·0048 | 0·0000 | 0·0000 | 0·0000 | 0·0000 | 0·0000 | 0·0000 | 0·0000 | 0·1440 |
| C17 | 0·0000 | 0·0000 | 0·0021 | 0·0000 | 0·0000 | 0·0000 | 0·0000 | 0·0000 | 0·0000 | 0·0000 | 0·0000 |
| C19 | 0·0000 | 0·0000 | 0·0000 | 0·0000 | 0·0000 | 0·0000 | 0·0000 | 0·2364 | 0·1570 | 0·0000 | 0·0864 |
| C20 | 0·0000 | 0·0000 | 0·0000 | 0·0000 | 0·0000 | 0·1862 | 0·0000 | 0·0000 | 0·0017 | 0·0000 | 0·0000 |
| C21 | 0·0000 | 0·0000 | 0·0149 | 0·0000 | 0·0315 | 0·0000 | 0·0005 | 0·0000 | 0·0000 | 0·0000 | 0·0433 |
| C22 | 0·0000 | 0·0000 | 0·0000 | 0·0000 | 0·0000 | 0·0000 | 0·0000 | 0·0000 | 0·0767 | 0·0000 | 0·0000 |
| C23 | 0·0186 | 0·0000 | 0·0019 | 0·0867 | 0·1058 | 0·0000 | 0·0173 | 0·0000 | 0·0000 | 0·0000 | 0·0073 |
| C24 | 0·0000 | 0·0000 | 0·0000 | 0·0000 | 0·0000 | 0·0000 | 0·0000 | 0·0000 | 0·0919 | 0·0000 | 0·0000 |
| C26 | 0·0000 | 0·0000 | 0·0000 | 0·1516 | 0·0413 | 0·0790 | 0·0000 | 0·0000 | 0·0112 | 0·0000 | 0·0269 |
| C27 | 0·0000 | 0·0000 | 0·0000 | 0·0000 | 0·0000 | 0·0000 | 0·2967 | 0·0000 | 0·0348 | 0·0000 | 0·0000 |
| C28 | 0·0000 | 0·0000 | 0·0000 | 0·0000 | 0·0000 | 0·0000 | 0·0000 | 0·0000 | 0·0000 | 0·5189 | 0·0000 |
| C29 | 0·0000 | 0·0000 | 0·0000 | 0·0000 | 0·0762 | 0·0000 | 0·0000 | 0·0000 | 0·0277 | 0·0000 | 0·0153 |
| C30 | 0·1780 | 0·0000 | 0·0000 | 0·3011 | 0·0000 | 0·0000 | 0·0000 | 0·0000 | 0·0000 | 0·0000 | 0·0192 |
| C31 | 0·0892 | 0·0237 | 0·0518 | 0·0000 | 0·1068 | 0·0000 | 0·0152 | 0·0202 | 0·0000 | 0·0000 | 0·0000 |
| C32 | 0·0000 | 0·0000 | 0·0000 | 0·0000 | 0·0000 | 0·0000 | 0·0098 | 0·0000 | 0·0000 | 0·0000 | 0·0891 |
| C33 | 0·0000 | 0·0000 | 0·0000 | 0·0000 | 0·0000 | 0·0414 | 0·0000 | 0·0000 | 0·0000 | 0·0788 | 0·0000 |
| C34 | 0·0000 | 0·0000 | 0·0000 | 0·0000 | 0·0000 | 0·0359 | 0·0000 | 0·0613 | 0·0000 | 0·0000 | 0·0000 |
| C36 | 0·0000 | 0·0047 | 0·1316 | 0·0000 | 0·0000 | 0·0000 | 0·0000 | 0·0000 | 0·0103 | 0·3427 | 0·0000 |
| C37 | 0·0000 | 0·0000 | 0·0000 | 0·0000 | 0·0000 | 0·0000 | 0·0000 | 0·0000 | 0·0000 | 0·0000 | 0·0000 |
| C38 | 0·0000 | 0·0028 | 0·0000 | 0·0000 | 0·0038 | 0·0651 | 0·0000 | 0·0012 | 0·0000 | 0·0000 | 0·0178 |
| C40 | 0·1033 | 0·0306 | 0·0000 | 0·0000 | 0·0000 | 0·0000 | 0·0637 | 0·0000 | 0·0447 | 0·0000 | 0·0000 |
| C41 | 0·0000 | 0·0000 | 0·0000 | 0·0000 | 0·0000 | 0·0000 | 0·0000 | 0·0000 | 0·0000 | 0·0000 | 0·0000 |
| C42 | 0·0069 | 0·0000 | 0·0000 | 0·0000 | 0·0000 | 0·0000 | 0·0152 | 0·0000 | 0·0000 | 0·0000 | 0·0000 |
| C44 | 0·0010 | 0·0000 | 0·0000 | 0·0000 | 0·0000 | 0·0000 | 0·0000 | 0·0000 | 0·0000 | 0·0000 | 0·0000 |
| C45 | 0·0000 | 0·0000 | 0·3320 | 0·0000 | 0·0000 | 0·0000 | 0·0000 | 0·0000 | 0·2066 | 0·0000 | 0·0170 |
| C46 | 0·0000 | 0·0000 | 0·0000 | 0·0000 | 0·0000 | 0·0000 | 0·0000 | 0·0000 | 0·0000 | 0·0000 | 0·0138 |
| C47 | 0·0000 | 0·0000 | 0·0000 | 0·0000 | 0·0000 | 0·0000 | 0·1150 | 0·0000 | 0·0003 | 0·0000 | 0·0000 |
| C49 | 0·0000 | 0·0000 | 0·0000 | 0·0000 | 0·0000 | 0·0000 | 0·0000 | 0·0000 | 0·0000 | 0·0000 | 0·0000 |
| C50 | 0·0000 | 0·0000 | 0·0065 | 0·0000 | 0·0454 | 0·0164 | 0·0000 | 0·0339 | 0·0000 | 0·0004 | 0·0050 |
| C51 | 0·0104 | 0·0214 | 0·0379 | 0·0000 | 0·0829 | 0·0000 | 0·0000 | 0·1159 | 0·0000 | 0·0000 | 0·0121 |
| C52 | 0·0000 | 0·0000 | 0·0000 | 0·0000 | 0·0000 | 0·0615 | 0·0000 | 0·0000 | 0·0002 | 0·0292 | 0·0000 |
| C54 | 0·1600 | 0·0000 | 0·0000 | 0·0000 | 0·0000 | 0·0000 | 0·0000 | 0·1076 | 0·0000 | 0·0000 | 0·0000 |
| C55 | 0·0000 | 0·0000 | 0·0115 | 0·2088 | 0·0000 | 0·0000 | 0·0000 | 0·0000 | 0·0189 | 0·0000 | 0·0274 |
| C56 | 0·1259 | 0·1590 | 0·0000 | 0·0000 | 0·0927 | 0·0000 | 0·0000 | 0·0000 | 0·0000 | 0·0000 | 0·0266 |
| C57 | 0·0000 | 0·0000 | 0·0171 | 0·0000 | 0·0000 | 0·0000 | 0·0120 | 0·0000 | 0·0314 | 0·0125 | 0·0000 |
| C58 | 0·0000 | 0·0000 | 0·0000 | 0·0000 | 0·0000 | 0·0000 | 0·0000 | 0·0000 | 0·0548 | 0·0000 | 0·0000 |
| C59 | 0·0000 | 0·0000 | 0·0000 | 0·0309 | 0·0000 | 0·0000 | 0·0425 | 0·0000 | 0·0000 | 0·0000 | 0·0000 |
| C60 | 0·0000 | 0·0256 | 0·0009 | 0·0000 | 0·0000 | 0·0000 | 0·0000 | 0·0138 | 0·0000 | 0·0000 | 0·0000 |
| C63 | 0·0263 | 0·0172 | 0·0000 | 0·0000 | 0·0000 | 0·0000 | 0·0000 | 0·0885 | 0·0000 | 0·0000 | 0·0000 |
| C65 | 0·0000 | 0·0000 | 0·0000 | 0·0000 | 0·0000 | 0·1044 | 0·0501 | 0·0000 | 0·0000 | 0·0000 | 0·0000 |
| C66 | 0·0000 | 0·0117 | 0·0000 | 0·0000 | 0·0000 | 0·0157 | 0·0408 | 0·0000 | 0·0000 | 0·0079 | 0·0000 |
| C67 | 0·0091 | 0·0054 | 0·0008 | 0·0000 | 0·2305 | 0·0000 | 0·0000 | 0·0000 | 0·0260 | 0·0000 | 0·2086 |
| C68 | 0·0000 | 0·0592 | 0·0000 | 0·0000 | 0·0000 | 0·0000 | 0·0000 | 0·0000 | 0·0756 | 0·0000 | 0·0000 |
| C69 | 0·0243 | 0·0458 | 0·0000 | 0·0000 | 0·0000 | 0·0184 | 0·0000 | 0·0000 | 0·0000 | 0·0000 | 0·0172 |
| C71 | 0·0000 | 0·0000 | 0·0057 | 0·0000 | 0·1829 | 0·0935 | 0·0000 | 0·0757 | 0·0000 | 0·0000 | 0·0000 |
| C72 | 0·0785 | 0·0000 | 0·0000 | 0·0470 | 0·0000 | 0·0000 | 0·0000 | 0·0000 | 0·0041 | 0·0000 | 0·0023 |
| C74 | 0·0969 | 0·0253 | 0·0000 | 0·0000 | 0·0000 | 0·0000 | 0·0000 | 0·0000 | 0·0027 | 0·0000 | 0·0000 |
| C76 | 0·0000 | 0·0000 | 0·0000 | 0·0000 | 0·0000 | 0·0000 | 0·0000 | 0·0000 | 0·0095 | 0·0000 | 0·0000 |
| C77 | 0·0000 | 0·0000 | 0·0000 | 0·0000 | 0·0000 | 0·2824 | 0·0000 | 0·0000 | 0·0000 | 0·0000 | 0·0000 |
| C78 | 0·0000 | 0·0000 | 0·0186 | 0·0000 | 0·0000 | 0·0000 | 0·0000 | 0·1672 | 0·0000 | 0·0096 | 0·0000 |
| C79 | 0·0000 | 0·0000 | 0·0000 | 0·0000 | 0·0000 | 0·0000 | 0·0000 | 0·0000 | 0·0000 | 0·0000 | 0·0000 |

**Supplementary Table S6.** Pre- and post-intervention dengue incidence rates and raw cases for each actual and synthetic control (SC) version of directly-intervened sites averaged across weeks over the entire observation period (EW 1 2014 – EW 25 2022), with corresponding standard deviations in parentheses. Incidence rates are calculated as the number of cases per 100,000 people in the population at risk of the site. Text in red indicates that that site did not pass validation criteria (i.e. visual inspection, RMSE checks, and/or placebo tests).

|  | **Dengue incidence** | | | | | | | |
| --- | --- | --- | --- | --- | --- | --- | --- | --- |
|  | **Pre-intervention** | | | | **Post-intervention** | | | |
|  | **Actual** | | **SC** | | **Actual** | | **SC** | |
| **Site** | **Incidence rate** | **Raw cases** | **Incidence rate** | **Raw cases** | **Incidence rate** | **Raw cases** | **Incidence rate** | **Raw cases** |
| W01 | 49.74 (89.48) | 1.99 (3.58) | 37.28 (30.59) | 1.49 (1.22) | 6.25 (16.22) | 0.25 (0.65) | 22.75 (28.60) | 0.91 (1.14) |
| W02 | 54.98 (96.41) | 0.86 (1.50) | 47.40 (34.15) | 0.74 (0.53) | 4.52 (16.46) | 0.07 (0.26) | 25.82 (35.31) | 0.40 (0.55) |
| W03 | 8.96 (13.36) | 0.77 (1.15) | 11.54 (7.96) | 1.00 (0.69) | 3.26 (6.68) | 0.28 (0.58) | 8.38 (7.45) | 0.72 (0.64) |
| W04 | 10.69 (18.10) | 0.91 (1.55) | 13.00 (8.06) | 1.11 (0.69) | 5.78 (12.43) | 0.49 (1.06) | 9.16 (7.60) | 0.78 (0.65) |
| W05 | 292.13 (468.57) | 2.19 (3.51) | 111.23 (125.91) | 0.83 (0.94) | 194.02 (388.02) | 1.46 (2.91) | 47.53 (103.15) | 0.36 (0.77) |
| W07 | 27.57 (35.10) | 2.76 (3.51) | 27.97 (21.04) | 2.80 (2.10) | 7.18 (14.00) | 0.72 (1.40) | 16.19 (16.38) | 1.62 (1.64) |
| W08 | 23.09 (32.51) | 1.39 (1.95) | 24.29 (18.58) | 1.46 (1.11) | 7.26 (14.19) | 0.44 (0.85) | 16.22 (18.05) | 0.97 (1.08) |
| W09 | 8.29 (15.38) | 1.12 (2.09) | 11.62 (9.25) | 1.58 (1.26) | 4.16 (9.06) | 0.56 (1.23) | 7.95 (9.37) | 1.08 (1.27) |
| W10 | 5.41 (8.54) | 0.49 (0.78) | 8.64 (5.04) | 0.79 (0.46) | 4.01 (9.41) | 0.37 (0.86) | 6.87 (5.71) | 0.63 (0.52) |
| W11 | 7.72 (14.09) | 0.44 (0.81) | 10.50 (6.91) | 0.60 (0.40) | 2.23 (6.75) | 0.13 (0.39) | 8.03 (7.36) | 0.46 (0.42) |
| W15 | 15.09 (30.89) | 0.52 (1.07) | 17.60 (10.79) | 0.61 (0.37) | 11.46 (33.49) | 0.40 (1.16) | 7.32 (8.78) | 0.25 (0.30) |
| W16 | 10.90 (32.87) | 0.45 (1.36) | 17.76 (15.40) | 0.74 (0.64) | 1.13 (5.94) | 0.05 (0.25) | 5.22 (5.78) | 0.22 (0.24) |
| W17 | 5.68 (16.56) | 0.14 (0.41) | 10.22 (6.30) | 0.25 (0.15) | 0.32 (3.61) | 0.01 (0.09) | 4.56 (3.68) | 0.11 (0.09) |
| W18 | 22.06 (36.07) | 2.21 (3.61) | 23.55 (17.35) | 2.36 (1.74) | 4.33 (8.35) | 0.43 (0.84) | 5.62 (5.48) | 0.56 (0.55) |
| W19 | 10.19 (17.44) | 0.57 (0.98) | 12.74 (8.27) | 0.71 (0.46) | 2.58 (7.36) | 0.14 (0.41) | 4.84 (4.19) | 0.27 (0.23) |
| W21 | 32.36 (32.77) | 4.49 (4.55) | 31.09 (20.40) | 4.31 (2.83) | 8.76 (11.91) | 1.22 (1.65) | 20.53 (15.70) | 2.85 (2.18) |
| W22 | 51.44 (36.87) | 6.03 (4.32) | 45.50 (21.01) | 5.33 (2.46) | 12.02 (16.72) | 1.41 (1.96) | 27.96 (19.79) | 3.28 (2.32) |
| W24 | 65.60 (82.80) | 3.69 (4.66) | 51.83 (39.44) | 2.92 (2.22) | 11.57 (20.65) | 0.65 (1.16) | 38.43 (42.40) | 2.16 (2.39) |
| W26 | 14.82 (54.26) | 0.14 (0.50) | 16.89 (13.59) | 0.16 (0.13) | 4.73 (27.50) | 0.04 (0.25) | 6.78 (8.92) | 0.06 (0.08) |
| W27 | 79.18 (126.39) | 1.55 (2.48) | 67.33 (62.49) | 1.32 (1.22) | 27.86 (65.73) | 0.55 (1.29) | 36.83 (60.27) | 0.72 (1.18) |

**Supplementary Table S7.** Pre- and post-intervention dengue incidence rates and raw cases for each actual and synthetic control (SC) version of spillover sites, averaged across weeks over the observation period (EW 1 2014 – EW 25 2022). The values in parentheses represent the standard deviation of these weekly averages. The incidence rates are calculated as the number of cases per 100,000 people in the population at risk of the site. Text in red indicates that that site did not pass validation criteria (i.e. visual inspection, RMSE checks, and/or placebo tests).

|  | **Dengue incidence** | | | | | | | |
| --- | --- | --- | --- | --- | --- | --- | --- | --- |
|  | **Pre-intervention** | | | | **Post-intervention** | | | |
|  | **Actual** | | **SC** | | **Actual** | | **SC** | |
| **Site** | **Incidence rate** | **Raw cases** | **Incidence rate** | **Raw cases** | **Incidence rate** | **Raw cases** | **Incidence rate** | **Raw cases** |
| C03 | 17.71 (28.60) | 0.89 (1.43) | 19.31 (13.30) | 0.97 (0.66) | 5.43 (12.18) | 0.27 (0.61) | 8.61 (8.88) | 0.43 (0.44) |
| C25 | 70.75 (123.41) | 1.13 (1.97) | 52.59 (41.36) | 0.84 (0.66) | 50.48 (88.99) | 0.81 (1.42) | 25.36 (27.35) | 0.41 (0.44) |
| C35 | 27.87 (51.18) | 1.07 (1.96) | 30.98 (23.26) | 1.19 (0.89) | 6.20 (17.09) | 0.24 (0.65) | 17.26 (19.35) | 0.66 (0.74) |
| C39 | 21.28 (45.37) | 0.83 (1.77) | 22.53 (14.86) | 0.88 (0.58) | 10.19 (20.32) | 0.40 (0.79) | 14.30 (16.34) | 0.56 (0.64) |
| C43 | 25.04 (38.17) | 0.85 (1.29) | 26.51 (18.21) | 0.90 (0.62) | 17.40 (36.83) | 0.59 (1.25) | 20.59 (31.36) | 0.70 (1.06) |
| C48 | 30.56 (87.71) | 0.59 (1.68) | 37.04 (28.00) | 0.71 (0.54) | 4.34 (19.58) | 0.08 (0.38) | 16.98 (18.33) | 0.33 (0.35) |
| C53 | 19.50 (41.46) | 0.76 (1.62) | 22.69 (23.78) | 0.88 (0.93) | 4.27 (13.92) | 0.17 (0.54) | 12.77 (19.85) | 0.50 (0.77) |
| C61 | 23.33 (42.76) | 0.58 (1.07) | 25.22 (19.81) | 0.63 (0.50) | 26.41 (56.11) | 0.66 (1.40) | 17.06 (26.11) | 0.43 (0.65) |
| C62 | 84.72 (120.00) | 0.85 (1.20) | 61.46 (48.93) | 0.61 (0.49) | 39.10 (83.92) | 0.39 (0.84) | 36.94 (42.76) | 0.37 (0.43) |
| C70 | 49.52 (91.33) | 0.57 (1.05) | 43.60 (31.97) | 0.50 (0.37) | 24.53 (61.52) | 0.28 (0.71) | 28.28 (31.08) | 0.33 (0.36) |
| C73 | 26.37 (92.57) | 0.40 (1.39) | 23.35 (22.51) | 0.35 (0.34) | 3.70 (18.32) | 0.06 (0.27) | 6.00 (7.92) | 0.09 (0.12) |

**Supplementary Table S8.** Protective effectiveness (PE) and absolute cases averted estimates for each directly-intervened using standard SCM (left, refer to Supplementary Methods) and SCM under partial interference (right). The PE values represent the percentage reduction in cases due to *Wolbachia*, while absolute cases averted are the number of cases prevented. Text in blue indicates sites that failed the RMSE check (i.e., RMSE > 100). Numbers in parenthesis represent lower and upper bounds for 95% confidence intervals, estimated using the bootstrapping procedure. Only sites that passed validation criteria (i.e. visual inspections, RMSE checks, placebo tests) were included in the calculation for aggregate PE and absolute cases averted.

|  | **Original SCM** | | | **SCM under partial interference** | | |
| --- | --- | --- | --- | --- | --- | --- |
| **Site** | **Protective effectiveness (%)** | **Absolute cases averted** | **RMSE** | **Protective effectiveness (%)** | **Absolute cases averted** | **RMSE** |
| W01 | 68·20 (66·99 - 69·31) | 83 (71 - 87) | 71·19 | 72.53 (71.40 - 73.61) | 97 (90 - 101) | 80·59 |
| W02 | 82·24 (81·37 - 82·79) | 36 (32 - 40) | 87·31 | 82.49 (81.62 - 83.10) | 40 (36 - 44) | 87·67 |
| W03 | 60·61 (56·31 - 61·81) | 59 (53 - 67) | 11·66 | 61.05 (56.91 - 62.26) | 59 (54 - 68) | 11·66 |
| W04 | 36·74 (34·08 - 37·89) | 35 (28 - 42) | 16·77 | 36.91 (34.37 - 38.02) | 35 (27 - 43) | 16·77 |
| W05 | -279·86 (-306·16 - -269·66) | -177 (-193 - -168) | 501·07 | -308.20 (-346.20 - -288.44) | -175 (-195 - -164) | 502·25 |
| W07 | 53·76 (52·98 - 55·22) | 132 (113 - 139) | 25·41 | 55.64 (55.10 - 57.16) | 138 (126 - 149) | 26·25 |
| W08 | 54·92 (53·91 - 56·22) | 68 (61 - 73) | 23·85 | 55.20 (54.15 - 56.50) | 69 (61 - 77) | 24·06 |
| W09 | 47·79 (44·61 - 61·69) | 77 (64 - 89) | 12·99 | 47.70 (44.54 - 61.69) | 75 (64 - 89) | 12·99 |
| W10 | 41·66 (41·00 - 43·12) | 29 (22 - 37) | 10·07 | 41.66 (41.00 - 43.12) | 29 (22 - 37) | 10·07 |
| W11 | 72·28 (71·78 - 73·23) | 37 (33 - 46) | 13·71 | 72.28 (71.78 - 73.23) | 37 (33 - 46) | 13·71 |
| W15 | -56·19 (-58·48 - -53·48) | -34 (-35 - -33) | 28·44 | -56.48 (-58.75 - -54.25) | -34 (-35 - -34) | 28·46 |
| W16 | 79·08 (78·34 - 79·63) | 13 (9 - 14) | 27·80 | 78.34 (77.56 - 79.05) | 9 (8 - 10) | 28·16 |
| W17 | 92·88 (92·66 - 93·14) | -1 (-1 - 0) | 16·72 | 93.01 (92.75 - 93.24) | 0 (-1 - 0) | 16·73 |
| W18 | 21·24 (19·94 - 23·30) | 2 (2 - 6) | 25·37 | 22.84 (21.50 - 24.92) | 8 (6 - 10) | 28·71 |
| W19 | 46·22 (45·94 - 51·08) | 1 (0 - 3) | 15·09 | 46.68 (46.64 - 51.69) | 3 (2 - 6) | 15·34 |
| W21 | 57·99 (56·36 - 67·82) | 413 (404 - 470) | 25·16 | 57.33 (55.76 - 67.49) | 392 (385 - 461) | 25·63 |
| W22 | 59·62 (58·86 - 61·11) | 555 (537 - 601) | 34·15 | 57.01 (56.29 - 58.63) | 512 (495 - 541) | 34·65 |
| W24 | 69·64 (69·18 - 70·72) | 359 (341 - 376) | 67·87 | 69.90 (69.42 - 71.15) | 363 (350 - 392) | 68·28 |
| W26 | 35·49 (32·46 - 37·42) | -3 (-4 - -3) | 52·00 | 30.28 (26.08 - 33.47) | -4 (-4 - -4) | 52·76 |
| W27 | 36·35 (34·65 - 38·36) | 43 (39 - 52) | 103·58 | 24.37 (22.11 - 27.50) | 20 (17 - 29) | 106·80 |
| **Aggregate** | **64·19 (59·41 - 66·40)** | **1834 (1788 - 1959)** | - | **64.35 (63.50 - 66.71)** | **1802 (1768 - 1930)** | - |

**Supplementary Table S9.** Protective effectiveness (PE) and absolute cases averted estimates for each spillover site using SCM under partial interference. The PE values represent the percentage reduction in cases due to *Wolbachia*, while absolute cases averted are the number of cases prevented. Text in blue indicates sites that failed the RMSE check (i.e., RMSE > 100). Numbers in parenthesis represent lower and upper bounds for 95% confidence intervals, estimated using the bootstrapping procedure. For spillover sites, the intervened site contributing to the spillover effect and the distance to the intervened site in metres are also shown. Only sites that passed the validation criteria (i.e. visual inspections, RMSE checks, placebo tests) were included in the calculation for aggregate PE and absolute cases averted.

| **Site** | **Protective effectiveness (%)** | **Absolute cases averted** | **RMSE** | **Spillover from** | **Distance to intervened site (m)** |
| --- | --- | --- | --- | --- | --- |
| C43 | 15.49 (13.60 - 17.57) | 3 (-3 - 10) | 32·82 | W21 | 473 |
| C35 | 64.07 (62.55 - 65.09) | 60 (56 - 64) | 42·06 | W01 | 1091 |
| C61 | -54.83 (-59.41 - -53.20) | -51 (-54 - -49) | 37·01 | W01 | 1368 |
| C39 | 28.72 (27.05 - 30.51) | 15 (10 - 19) | 41·12 | W01 | 1399 |
| C48 | 74.43 (73.54 - 75.06) | 29 (26 - 31) | 79·31 | W01 | 1440 |
| C62 | -5.84 (-7.82 - -1.74) | -17 (-19 - -15) | 113·25 | W03, W19 | 624, 506 |
| C25 | -99.09 (-110.39 - -92.79) | -73 (-78 - -72) | 113·48 | W05 | 919 |
| C53 | 66.54 (65.23 - 67.17) | 35 (33 - 41) | 32·14 | W07 | 1388 |
| C70 | 13.28 (10.52 - 15.17) | -8 (-9 - -3) | 83·88 | W08 | 824 |
| C03 | 36.95 (35.75 - 38.25) | 13 (10 - 14) | 24·49 | W15 | 263 |
| C73 | 38.25 (36.04 - 39.94) | -5 (-5 - -5) | 85·32 | W18 | 601 |
| **Aggregate** | 37.69 (36.45 - 38.49) | 115 (104 - 132) | - | - | - |

**Supplementary Table S10.** Protective effectiveness (PE) estimates from in-time placebo tests for each directly-intervened site with their accompanying RMSE. The in-time placebo test assesses the ability of the synthetic control model to distinguish actual intervention effects from pre-intervention trends. It accomplishes this by assigning a hypothetical intervention start time earlier than the actual intervention start and analysing the data as if the intervention had occurred. Text in red indicates that the intervention site exhibited a protective effectiveness (PE) larger than its actual PE (refer to Supplementary Table S4) prior to the start of the intervention, thereby failing to meet the criteria of the in-time placebo test. Text in blue indicates that that site’s RMSE > 100. Only sites that passed the validation criteria were included in the calculation for aggregate PE.

|  | **Original SCM** | | **SCM under partial interference** | |
| --- | --- | --- | --- | --- |
| **Site** | **Protective effectiveness (%)** | **RMSE** | **Protective effectiveness (%)** | **RMSE** |
| W01 | -52·19 | 78·09 | 5·34 | 78·09 |
| W02 | -197·40 | 63·66 | -218·84 | 70·55 |
| W03 | 36·12 | 11·17 | 26·78 | 10·78 |
| W04 | 76·56 | 17·06 | 80·23 | 15·78 |
| W05 | -464·34 | 348·05 | -375·03 | 383·04 |
| W07 | -115·92 | 14·70 | -162·18 | 16·70 |
| W08 | -29·18 | 19·72 | -59·33 | 19·98 |
| W09 | 20·87 | 11·43 | 34·71 | 11·70 |
| W10 | 39·18 | 8·60 | 53·57 | 9·01 |
| W11 | 8·92 | 10·62 | 38·14 | 13·48 |
| W15 | 16·09 | 24·46 | 39·28 | 27·45 |
| W16 | 3·03 | 16·56 | -120·51 | 15·94 |
| W17 | 52·47 | 15·04 | 6·14 | 14·81 |
| W18 | 46·75 | 27·62 | -8·48 | 30·32 |
| W19 | -33·36 | 11·78 | 39·92 | 15·69 |
| W21 | -11·15 | 26·80 | 50·15 | 26·78 |
| W22 | -69·71 | 23·67 | -52·71 | 29·38 |
| W24 | -146·27 | 61·27 | -22·05 | 69·49 |
| W26 | -44·80 | 33·23 | 64·77 | 54·22 |
| W27 | -116·40 | 87·86 | -169·77 | 92·53 |
| **Aggregated** | **-55·42** | - | **-40·90** | - |

**Supplementary Table S11.** Protective effectiveness (PE) estimates from in-time placebo tests for each spillover site with their accompanying RMSE. Text in red indicates that the intervention site exhibited a protective effectiveness (PE) larger than its actual PE (refer to Supplementary Table S4) prior to the start of intervention, thereby failing to meet the criteria of the in-time placebo test. Text in blue indicates that that site’s RMSE > 100. Only sites that passed the validation criteria were included in the calculation for aggregate PE.

| **Spillover placebo site** | **Protective effectiveness (%)** | **RMSE** |
| --- | --- | --- |
| C43 | 52·22 | 33·17 |
| C35 | 15·20 | 38·20 |
| C61 | 3·11 | 33·27 |
| C39 | 25·61 | 44·95 |
| C48 | 94·42 | 92·89 |
| C62 | -264·97 | 91·83 |
| C25 | -1·56 | 112·99 |
| C53 | 57·36 | 37·25 |
| C70 | -41·89 | 72·22 |
| C03 | -16·27 | 22·03 |
| C73 | 86·02 | 98·56 |
| **Aggregated** | **-10·49** | - |

**Supplementary Table S12.** Protective effectiveness (PE) estimates for each placebo-intervention site (i.e. pure controls) and placebo-spillover site with their accompanying RMSE. The in-space placebo test examines whether the observed intervention effects are localised to the actual intervention sites. It achieves this by designating control sites as 'treated' in a counterfactual analysis and confirms that placebo 'treatments' do not yield significant effects. Text highlighted in red indicates that the intervention site had a PE larger than the actual directly-intervened site aggregated PE of 64.35%, thus failing the in-space placebo test. Text in blue indicates that the site’s RMSE exceeds 100. Text in blue indicates that that site’s RMSE exceeds 100. For spillover sites, the intervened site contributing to the spillover effect and the distance to the intervened site in metres are also shown. Only sites that passed the validation criteria were included in the calculation for aggregate PE.

|  | **Original SCM** | | **SCM under partial interference** | |
| --- | --- | --- | --- | --- |
| **Site** | **Protective effectiveness (%)** | **RMSE** | **Protective effectiveness (%)** | **RMSE** |
| C01 | 55·57 | 48·00 | 44·92 | 48·78 |
| C02 | 28·41 | 14·56 | 24·09 | 15·21 |
| C04 | 18·64 | 22·10 | 31·78 | 65·65 |
| C05 | 41·26 | 64·57 | -0·07 | 9·08 |
| C06 | -0·07 | 9·08 | -37·36 | 23·23 |
| C07 | -33·26 | 22·73 | 10·69 | 10·15 |
| C08 | 10·69 | 10·15 | -5·00 | 11·90 |
| C09 | -2·88 | 11·88 | 12·46 | 60·28 |
| C10 | 19·88 | 59·33 | 52·99 | 10·27 |
| C11 | 53·23 | 10·23 | 45·62 | 70·62 |
| C12 | 46·04 | 70·51 | 62·75 | 29·31 |
| C13 | 62·75 | 29·31 | -4·75 | 36·50 |
| C14 | -3·78 | 35·10 | 58·07 | 11·63 |
| C15 | 58·16 | 11·59 | -38·38 | 23·46 |
| C16 | -38·38 | 23·46 | 6·94 | 28·21 |
| C17 | 7·11 | 28·16 | -30·63 | 26·82 |
| C19 | -32·12 | 26·82 | -49·29 | 11·84 |
| C20 | -44·50 | 11·82 | 61·47 | 26·20 |
| C21 | 62·41 | 25·93 | 16·71 | 82·99 |
| C22 | 16·71 | 82·99 | 1·87 | 16·34 |
| C23 | -1·76 | 15·98 | -50·88 | 63·86 |
| C24 | -83·60 | 61·70 | 33·92 | 9·79 |
| C26 | 33·03 | 9·72 | 12·93 | 124·95 |
| C27 | -10·72 | 113·06 | 50·40 | 65·24 |
| C28 | 2·73 | 121·15 | 46·21 | 27·63 |
| C29 | 44·32 | 63·61 | -51·56 | 49·18 |
| C30 | 41·44 | 25·41 | 68·68 | 10·59 |
| C31 | -32·74 | 47·49 | -67·98 | 141·10 |
| C32 | 69·30 | 10·54 | -6·33 | 43·83 |
| C33 | -67·98 | 141·10 | -47·88 | 62·24 |
| C34 | -4·36 | 43·78 | -66·84 | 36·48 |
| C36 | -48·43 | 55·85 | -0·16 | 26·61 |
| C37 | -62·91 | 36·35 | 29·75 | 18·69 |
| C38 | 48·21 | 38·83 | -144·34 | 275·92 |
| C40 | 0·66 | 26·61 | 36·73 | 34·68 |
| C41 | 28·56 | 18·66 | 11·33 | 12·73 |
| C42 | -144·34 | 275·92 | 44·63 | 32·47 |
| C44 | 17·25 | 22·17 | 41·08 | 23·55 |
| C45 | 32·76 | 34·29 | 40·35 | 9·97 |
| C46 | 7·81 | 12·56 | 25·88 | 23·50 |
| C47 | 46·04 | 31·04 | 45·05 | 10·65 |
| C49 | 15·26 | 32·56 | 33·59 | 40·69 |
| C50 | 39·72 | 23·48 | -421·42 | 236·15 |
| C51 | 39·78 | 9·94 | 36·06 | 96·17 |
| C52 | 26·20 | 23·49 | -3·43 | 152·16 |
| C54 | 43·57 | 10·52 | 16·14 | 41·19 |
| C55 | 87·10 | 104·46 | 13·17 | 79·32 |
| C56 | 33·59 | 40·69 | -27·17 | 39·69 |
| C57 | -407·13 | 235·97 | 24·14 | 41·90 |
| C58 | 35·08 | 96·02 | -11·77 | 38·71 |
| C59 | -3·43 | 152·16 | 34·59 | 16·62 |
| C60 | 76·35 | 39·83 | -91·71 | 25·06 |
| C63 | 26·77 | 39·63 | -62·28 | 109·94 |
| C65 | -8·30 | 71·61 | 10·73 | 56·12 |
| C66 | -30·90 | 36·22 | 45·98 | 118·58 |
| C67 | 19·74 | 40·06 | -9·73 | 60·68 |
| C68 | -12·88 | 38·60 | -18·94 | 45·11 |
| C69 | 33·02 | 16·40 | 21·65 | 138·47 |
| C71 | -91·71 | 25·06 | -141·81 | 132·02 |
| C72 | -29·62 | 32·95 | -8·24 | 48·30 |
| C74 | -136·91 | 97·20 | -12·25 | 28·35 |
| C76 | -44·48 | 109·61 | -19·72 | 20·04 |
| C77 | 33·45 | 53·60 | -127·71 | 22·16 |
| C78 | 48·94 | 118·01 | -32·62 | 28·73 |
| C79 | -10·24 | 59·39 | 23·35 | 10·93 |
| C43 | -34·70 | 44·47 | - | - |
| C35 | 22·09 | 138·42 | - | - |
| C61 | -105·52 | 57·48 | - | - |
| C39 | -98·36 | 130·51 | - | - |
| C48 | -24·23 | 47·58 | - | - |
| C62 | 22·00 | 79·51 | - | - |
| C25 | -6·70 | 27·02 | - | - |
| C53 | -22·25 | 19·86 | - | - |
| C70 | -123·81 | 21·53 | - | - |
| C03 | -30·48 | 27·55 | - | - |
| C73 | 23·35 | 10·93 | - | - |
| **Aggregated** | **4·46** | - | **6·73** | - |

**Supplementary Table S13.** Protective effectiveness (PE) estimates for each placebo-intervention site placebo-spillover site with their accompanying RMSE. Text highlighted in red indicates that the intervention site had a PE larger than the actual spillover site aggregated PE of 37.69%, thus failing the in-space placebo test. Text in blue indicates that the site’s RMSE exceeds 100. Text in blue indicates that that site’s RMSE exceeds 100. The placebo-intervened site contributing to the placebo spillover effect and the distance to the placebo-intervened site in metres are also shown. Only sites that passed validation criteria were included in the calculation for aggregate PE and absolute cases averted.

| **Spillover placebo site** | **Protective effectiveness (%)** | **RMSE** | **Spillover from** | **Distance from placebo intervened site (m)** | |
| --- | --- | --- | --- | --- | --- |
| C20 | 61·47 | 26·20 | C09 | | 515 |
| C11 | 45·62 | 70·62 | C13 | | 1302 |
| C12 | 62·75 | 29·31 | C14 | | 1212 |
| C17 | -38·14 | 26·92 | C15 | | 259 |
| C13 | -4·78 | 37·14 | C21 | | 388 |
| C46 | 25·88 | 23·50 | C26 | | 1106 |
| C37 | 29·15 | 18·83 | C26 | | 563 |
| C42 | 46·64 | 32·52 | C28 | | 146 |
| C24 | 33·92 | 9·79 | C29 | | 1411 |
| C38 | -144·34 | 275·92 | C31 | | 602 |
| C71 | -141·81 | 132·02 | C40 | | 857 |
| C69 | 21·65 | 138·47 | C40 | | 608 |
| C67 | -13·87 | 60·44 | C41 | | 1300 |
| C63 | -62·28 | 109·94 | C47 | | 498 |
| C59 | 42·90 | 16·93 | C47 | | 976 |
| C71 | -139·99 | 132·03 | C49 | | 1255 |
| C36 | -6·97 | 27·43 | C49 | | 548 |
| C56 | -27·17 | 39·69 | C50 | | 617 |
| C24 | 33·46 | 10·41 | C55 | | 320 |
| C66 | 45·98 | 118·58 | C60 | | 1337 |
| C59 | 34·59 | 16·62 | C63 | | 979 |
| C40 | 38·90 | 34·85 | C65 | | 38 |
| C71 | -143·12 | 133·48 | C65 | | 886 |
| C69 | 21·65 | 138·47 | C65 | | 643 |
| C27 | 50·56 | 65·25 | C67 | | 344 |
| C36 | -2·96 | 26·75 | C69 | | 1027 |
| C69 | 21·65 | 138·47 | C71 | | 324 |
| C36 | -15·95 | 27·16 | C71 | | 707 |
| C30 | 68·68 | 10·59 | C72 | | 1489 |
| C47 | 44·69 | 10·66 | C74 | | 567 |
| C63 | -62·28 | 109·94 | C74 | | 984 |
| C59 | 42·90 | 16·93 | C74 | | 843 |
| C63 | -62·28 | 109·94 | C77 | | 1397 |
| **Aggregated** | **26·34** | - | - | | - |

**Supplementary Figure S1.** Observed (in grey) and synthetic control (in red) for all directly-intervened and spillover sites in the baseline synthetic control model across the whole study period. The start of *Wolbachia*-releases (intervention) or spillover (start of intervention at the closest directly-intervened site to spillover site) is indicated by the black dotted vertical line.

Dengue

incidence

rate

Year of study

Dengue

incidence

rate

Year of study

Year of study

Dengue

incidence

rate

Dengue

incidence

rate

Year of study

Dengue

incidence

rate

Year of study

Dengue

incidence

rate

Year of study

**Supplementary Figure S2.** Distribution of aggregated protective effectiveness (%) derived from permutation tests for direct protective effectiveness (PE) (left) and spillover PE (right), where intervention and control towns were randomly reassigned 1,000 times. In each permutation, spillover sites were defined based on their proximity to randomly assigned control towns, and synthetic controls were re-estimated to compute direct and spillover PEs. The actual aggregated direct PE (61.21%) and aggregated spillover PE (37.69%) are indicated by a dotted vertical line. Actual aggregated PEs are considered significant at the 5% level if they exceed 95% of the permutation PEs. The aggregated direct PE exceeded 100.0% (1000/1000) of permutation PEs, and the aggregated spillover PE exceeded 95.2% (952/1000) of permutation PEs.

**References**

1. Hoffmann, A. A. *et al.* Introduction of *Aedes aegypti* mosquitoes carrying *w*AlbB *Wolbachia* sharply decreases dengue incidence in disease hotspots. *iScience* **27**, 108942 (2024).

2. Nazni, W. A. *et al.* Establishment of *Wolbachia* Strain wAlbB in Malaysian Populations of Aedes aegypti for Dengue Control. *Current Biology* **29**, 4241-4248.e5 (2019).

3. Jasper, M., Schmidt, T. L., Ahmad, N. W., Sinkins, S. P. & Hoffmann, A. A. A genomic approach to inferring kinship reveals limited intergenerational dispersal in the yellow fever mosquito. *Molecular Ecology Resources* **19**, 1254–1264 (2019).

4. Abadie, A., Diamond, A. & Hainmueller, J. Synthetic Control Methods for Comparative Case Studies: Estimating the Effect of California’s Tobacco Control Program. *Journal of the American Statistical Association* **105**, 493–505 (2010).
